# One health perspectives on pharmaceutical and clinical effluents driving multidrug resistance in aquatic ecosystems: a comprehensive review

**DOI:** 10.3389/fsysb.2026.1890380

**Published:** 2026-10-14

**Authors:** Anshnu Tyagi, Minakshi Rajput, Sudhanshu Mishra

**Affiliations:** 1 School of Forensic Sciences, National Forensic Sciences University, Delhi, India; 2 Faculty of Forensic Science, Department of Chemistry, School of Physical Sciences, DIT University, Dehradun, Uttarakhand, India

**Keywords:** antibiotic resistance genes, antimicrobial resistance, aquatic ecosystem, One Health, pharmaceutical and clinical waste, sustainable development goals

## Abstract

Antimicrobial resistance (AMR) has emerged as one of the most pressing global health challenges, yet the contribution of environmental reservoirs to the spread of multidrug resistance (MDR) remains insufficiently integrated into mainstream discussions. This systematic review synthesizes evidence from 167 peer-reviewed studies published between 2000 and 2026 to examine how pharmaceutical manufacturing effluents and clinical waste drive the dissemination of antibiotic resistance genes (ARGs) and multidrug-resistant bacteria in aquatic ecosystems. The literature was systematically identified and selected from major scientific databases using predefined inclusion and exclusion criteria. The reviewed evidence consistently shows that aquatic environments receiving inadequately treated pharmaceutical and healthcare waste which creates environmental hotspots characterized by elevated antibiotic residues, mobile genetic elements, and resistant microbial communities. Sub-inhibitory antibiotic concentrations, biofilm-mediated horizontal gene transfer and quorum-sensing dynamics collectively accelerate resistance gene dissemination beyond what direct selective killing alone would predict. Heavy metals, including copper, zinc, mercury, cadmium, and arsenic, further reinforce resistance through co-selection mechanisms, sustaining clinically important determinants such as *blaNDM-1*, *blaKPC*, and *mcr-1* even in environments with limited antibiotic exposure. The review also highlights the role of non-pathogenic environmental bacteria as genetic reservoirs that facilitate the movement of resistance traits into pathogenic microorganisms. Metagenomic evidence confirms bidirectional exchange of resistance determinants between environmental and clinical bacterial populations, firmly situating the environmental resistome within a One Health framework. Despite growing evidence, important knowledge gaps remain regarding environmental-to-clinical transmission pathways, long-term ecological impacts, and the effectiveness of current mitigation strategies under diverse environmental conditions. Future research should prioritize longitudinal surveillance, multi-omics and systems biology approaches, metagenomic resistome profiling, and machine-learning-based predictive frameworks to better understand resistance dynamics across interconnected ecosystems. The findings support the integration of ARG monitoring into environmental management programs and underscore the need for advanced wastewater treatment technologies, harmonized discharge regulations, and coordinated One Health policies to curb the environmental amplification of antimicrobial resistance. These insights also contribute directly to achieving multiple Sustainable Development Goals, particularly SDG 1, 2, 3, 6, 9, 12, and 17 by fostering sustainable and integrated approaches to AMR mitigation.

## 1 Introduction

The rise and proliferation of multidrug resistance (MDR) pathogens represent one of the biggest threats to public health in the form of AMR being linked to hundreds of thousands of deaths annually and immense clinical, economic, and societal burden (Murray et al., 2022). The discussion around AMR for several decades was focused mainly on its origins in human, veterinary, and agricultural sectors due to improper and excessive use of antibiotics (O’Neill, 2016; Tiseo et al., 2020). Yet this understanding overlooks another aspect of the AMR problem which receives increasing attention and documentation–dissemination of drug resistance via discharge of pharmaceutical effluents, hospital wastewater, and medical wastes (Michael et al., 2013; Singer et al., 2016). Emerging information suggests that exposure to contaminated water and soil represents an important environmental pathway for the emergence and amplification of AMR through providing optimal ecological niches for coexistence and horizontal transmission of genes and bacteria (Wellington et al., 2013; Larsson and Flach, 2022). Crucially, acquired antibiotic resistance genes in such an environment have the potential to be introduced back into human beings through drinking water, irrigation systems, food chains, occupational exposure, and improperly treated wastewater. Such practices will create a vicious cycle that makes it difficult to control infections and limit their therapeutic effectiveness (WHO, 2024; Ragueh et al., 2025). Though there has been tremendous progress in the study of AMR, the literature still appears disjointed due to its wide coverage of various scientific fields including environmental microbiology, waste management, clinical epidemiology, and policy studies. For instance, while several studies concentrate on hospital wastes, pharmaceutical production effluent, wastewater treatment, and antibiotic resistance mechanism individually, few examine the same within the context of one health (Borsetto et al., 2025; Lamba et al., 2018). This gap results in inadequate information on how resistance from waste material is developed, spread, enhanced, and ultimately redistributed back into humans and animals. What needed is an all-encompassing review on how waste material develops resistance, what pathways are used to enhance the resistance, how exposure occurs, and the regulatory gaps that exist and contribute to the development and spread of the resistance. The review seeks to fill this gap by focusing on how aquatic ecosystems act as conduits for MDR bacteria, especially as related to pharmaceutical effluents and clinical waste as overlooked contributors to AMR spread. By incorporating an environmental, clinical, and regulatory perspective, the review aims at bringing out the wider public health, ecological, and developmental relevance of AMR spread through waste, particularly in relation to the SDGs, wastewater monitoring, integrated waste management, and antibiotic lifecycle regulation. In doing so, it reframes MDR not only as a biomedical challenge but also as an environmental pollution crisis requiring coordinated intervention across healthcare, industry, and environmental regulation.

### 1.1 Understanding antibiotics and the rise of resistance

#### 1.1.1 Antibiotics

The discovery by Alexander Fleming of penicillin in 1928 is regarded as a groundbreaking event in medicine, showing that some microorganisms may inhibit the growth of bacteria (Aminov, 2010). Waksman defined antibiotics as microbial products that act on other microorganisms by suppressing or killing them, playing an essential role in the treatment of infectious diseases (Davies and Davies, 2010). Antibiotics operate by interfering with crucial cellular functions such as the biosynthesis of the cell wall, protein synthesis, DNA replication, or metabolic processes and thus inhibit bacteria or kill them (Kohanski et al., 2010; Laxminarayan et al., 2013). The advent of antibiotics dramatically decreased the mortality from bacterial infections and led to major changes in both medicine and agriculture. However, the overuse and misuse of antibiotics have driven the emergence of drug-resistant bacteria. Resistance evolves among bacteria but not in humans, and it can be transmitted to other organisms, facilitating the rapid spread of resistance determinants (Zellweger et al., 2017; Holmes et al., 2016). As a result, antibiotic resistance, or more widely antimicrobial resistance, is now considered a serious threat to all medical advancements made in the past century.

#### 1.1.2 Antibiotic resistance

Antibiotic resistance refers to the ability of bacteria to continue living, multiplying, or surviving in the presence of antimicrobial drugs designed to kill or stop them from developing. This represents a serious threat to global health and extends beyond the boundaries of the clinical setting to include other communities around the world (Chen et al., 2023; Global Antibiotic Resistance Surveillance Report, 2025). With the development of resistance, fewer options, expensive, and ineffective treatments may follow, leading to complications, ineffective treatments, and fatalities (Murray et al., 2022; Tacconelli et al., 2018). Further difficulties arise as a result of the emergence of multidrug resistance, where bacteria demonstrate resistance to multiple types of antibiotics at once, thus reducing treatment alternatives (Watts et al., 2017; Naghavi et al., 2024). Due to the rapid evolution of bacteria, resistance can be acquired at a fast pace, especially when the antibiotic usage in the environment is high and there is little government regulation (Laxminarayan et al., 2013; Holmes et al., 2016). The rising rate of antibiotic use, especially in developing countries, has contributed further to this problem, making a need for an interdisciplinary approach toward addressing AMR more relevant than ever before (Van Boeckel et al., 2015; Klein et al., 2024). International independent analyses still consider antimicrobial resistance one of the greatest public health threats of our time, with millions of fatalities and financial damages expected if things keep going the same way (O'Neill, 2016; Naghavi et al., 2024).

## 2 Methodology

The present review followed a systematic and comprehensive approach to identify and evaluate published evidence on AMR, MDR, pharmaceutical and healthcare waste, environmental resistome, HGT, and the One Health framework. Methodology followed a systematic search and screening framework designed to capture both fundamental and application-oriented relevant studies (Shaheen et al., 2026). Selected articles were critically evaluated and integrated to provide a coherent understanding of the interconnections among these factors and to elucidate the complex pathways driving the emergence, dissemination, and persistence of antimicrobial resistance and their implications for public, animal, and environmental health. The scientific literature has been gathered from many online international databases like Google Scholar, PubMed, Science Direct, Scopus, Web of Science, and ResearchGate etc. The review focused on peer-reviewed articles published in English within two separate time frames: 2000–2015 (45 articles) and 2016–2026 (122 articles) reflecting the rapid development and research in the relevant field. Search strings conducted employing various combinations of specific keywords and Boolean operators such as antimicrobial resistance, multidrug resistance, pharmaceutical effluent, clinical waste, aquatic resistome, horizontal gene transfer, biofilm, One Health etc. After conducting the title, abstract, and full-text review, inclusion and exclusion criteria for the selection of the studies to be included in the present review have been decided. The inclusion criteria include, peer-reviewed research that looks into environmental, clinical, molecular, or public health aspects of AMR; peer-reviewed papers that discuss drug effluents, medical waste, or aquatic ecosystems as vectors for AMR/ARG spread; and peer-reviewed research that discusses HGT, MGEs, biofilm-related resistance, and co-selection, and One health. The exclusion criteria include, duplicates, irrelevant literature not pertinent to the focus of the study; publications having ambiguous or insufficient data; non-peer-reviewed items such as gray literature, dissertations, abstracts; and inaccessible literature. According to the aforementioned selection criteria, a sum of 1,380 articles have been identified initially via electronic databases and reviewed. 1,213 articles did not meet the inclusion criteria so that excluded and finally 167 articles are selected to be included in the present study as they met all inclusion criteria and containing the maximum relevant information. A stepwise flow diagram illustrating literature searches, screening, inclusion/exclusion process of the articles is presented in Figure 1.

**FIGURE 1:**
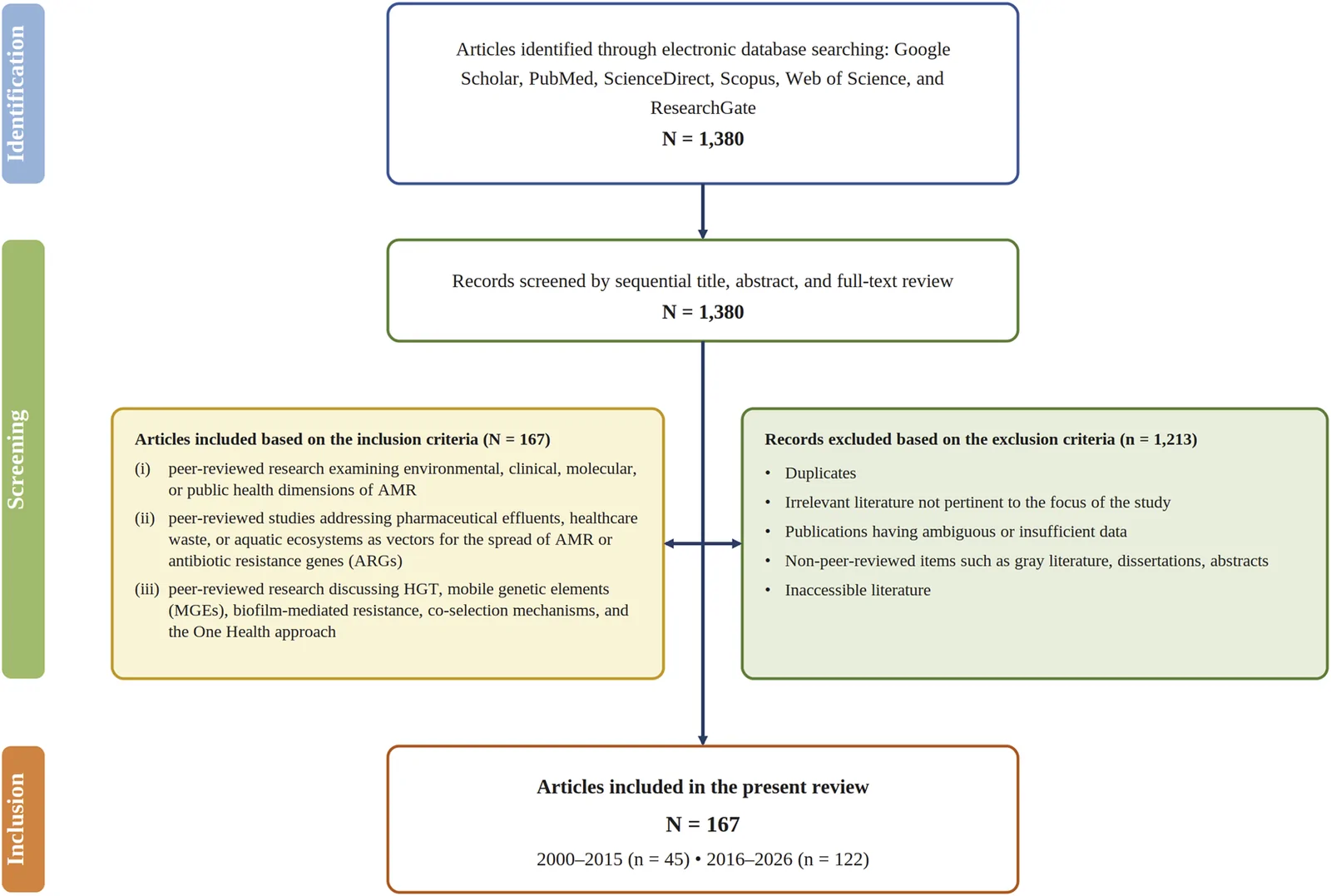
Overview of the systematic review methodology, including literature identification across electronic databases, sequential screening, application of predefined inclusion and exclusion criteria, and final selection of studies for evidence synthesis.

## 3 Pathways contributing to the emergence and spread of antibiotic resistance

### 3.1 Human and environmental factors in the spread of resistance

AMR is profoundly influenced by environmental interactions and cannot be viewed solely as a biological phenomenon. High antibiotic exposure in hospitals, pharmaceutical manufacturing facilities, and wastewater treatment plants exerts selective pressure on microorganisms, driving the evolution and dissemination of resistant strains (Adegoke, et al., 2016). The widespread presence of mobile genetic elements (MGEs) integrons, transposons, and plasmids further accelerates the horizontal transfer of resistance genes across bacterial species, amplifying the public-health threat (Michael et al., 2013; Naghavi et al., 2024). Despite advances in antibiotic discovery, unregulated production, misuse, and poor waste management continue to create conditions conducive to resistance. Hospital infections such as cholera, typhoid, and multidrug-resistant tuberculosis (MDR-TB) are increasingly difficult to treat, as highlighted by the WHO Global Tuberculosis Report (2024). This persistence stems from inadequate infection-control practices, improper antibiotic use, and insufficient oversight of pharmaceutical and healthcare waste. Improperly treated effluents containing antibiotic residues, biological waste, and contaminated clinical materials are discharged into the environment, where they contaminate soil and aquatic systems, perpetuating cycles of microbial adaptation and resistance (Tacconelli et al., 2018; La Rosa et al., 2025). Selective pressure remains a primary evolutionary force in this process. The indiscriminate use of antibiotics in human medicine, veterinary care, and livestock production provides resistant strains with a survival advantage over susceptible ones (Browne et al., 2021). Every unnecessary prescription or subtherapeutic dose acts as a microevolutionary experiment, promoting mutations and gene acquisition that enhance bacterial persistence (Murray et al., 2022; Tiseo et al., 2020). Environmental dissemination intensifies when antibiotics escape regulated settings. Improper disposal of unused drugs, untreated hospital effluents, and human or animal waste collectively enrich environmental reservoirs of resistance. These low-dose, chronic exposures exert continuous selective pressure that enables bacteria in natural ecosystems to acquire and retain resistance determinants (Larsson et al., 2018; Berglund, 2015). At the molecular level, mobile genetic elements play a central role in this adaptability. Plasmids (self-replicating DNA molecules) carry clusters of resistance genes and are readily transferred through conjugation, as seen in *E. coli* IncF plasmids harboring *bla*CTX-M genes that confer cephalosporin resistance (Brockhurst et al., 2019; Castaneda-Barba et al., 2024). Transposons, or “jumping genes,” facilitate the insertion of resistance genes into new genomic contexts, stabilizing them within bacterial lineages; for instance, Tn3 family transposons spread ampC β-lactamase genes, increasing penicillin and cephalosporin resistance (Partridge et al., 2018; Wang et al., 2023). Integrons function as gene-capture systems that assemble and express resistance cassettes particularly Class 1 integrons, which are prevalent in Gram-negative pathogens and contribute to multidrug resistance (Ruiz-Martínez et al., 2011; Burch et al., 2014). The detection of these elements in hospital drainage, livestock effluents, urban wastewater, and even soil microbiota demonstrates that resistance is not confined to healthcare settings but is part of an interconnected ecological crisis (Karkman et al., 2018; Alem et al., 2025). Addressing these pathways demands integrated interventions ranging from enhanced diagnostics and stewardship programs to stringent waste management policies. Without decisive global action, the relentless spread of resistance threatens to erode the foundations of modern medicine, rendering even routine infections increasingly untreatable (Laxminarayan et al., 2013; Naghavi et al., 2024).

### 3.2 Genetic basis for resistance

The emergence of antibiotic resistance lies on the platform of two generalized genetic mechanisms, namely, intrinsic and acquired resistance. Both are implicated in the mounting ineffectiveness of most first-line antibiotics in hospital and environmental circumstances (Blair et al., 2015; Chen et al., 2023).

#### 3.2.1 Intrinsic resistance

Some bacteria have inherent attributes that naturally resist certain antibiotics regardless of previous exposure. This intrinsic resistance (Figure 2a) is simply already present in the biology of the organism, usually as a result of evolutionary processes that occurred long before the clinical use of antibiotics. For example, *Pseudomonas aeruginosa*, a frequently encountered opportunistic pathogen, has intrinsic resistance to various antibiotic classes due to its low outer membrane permeability and highly efficient efflux pump mechanisms that remove antibiotics from the bacterial cytoplasm before they can have an effect (Li et al., 2015; Lister et al., 2009; Mazza et al., 2025). Likewise, *Enterococcus faecalis* naturally resists cephalosporins due to its low-affinity penicillin-binding proteins (Pbps), which decrease the effectiveness of β-lactam antibiotics (Nelson et al., 2024). Additionally, penicillin cannot act on organisms such as *Mycoplasma pneumoniae* that lack a typical peptidoglycan cell wall, its target, thereby demonstrating another type of intrinsic resistance (Halawa et al., 2024; Keller and Dörr, 2023).

**FIGURE 2:**
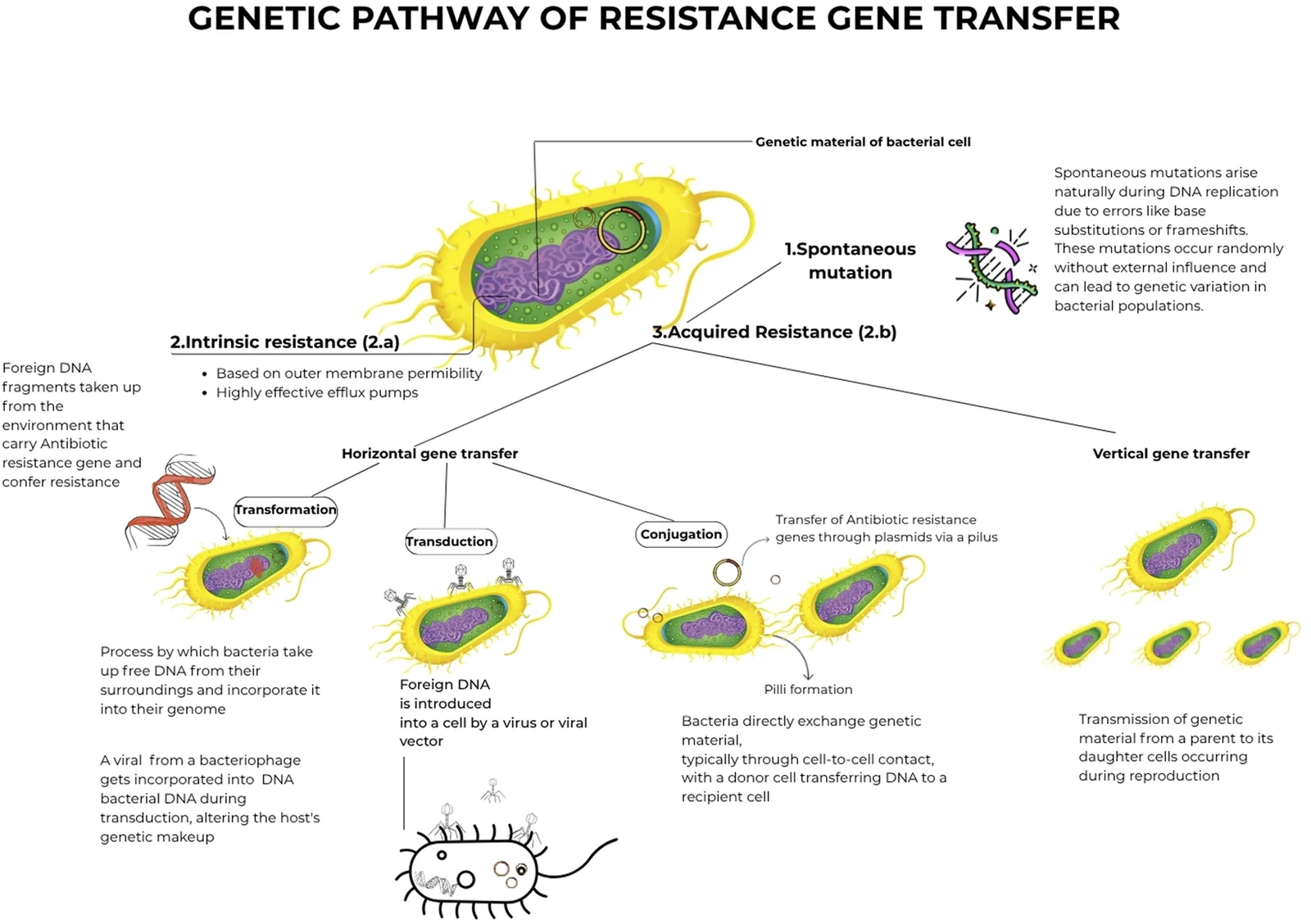
Genetic pathways contributing to antibiotic resistance in bacteria, including intrinsic and extrinsic mechanisms. Intrinsic resistance is naturally encoded within the bacterial chromosome and passed through vertical gene transfer (VGT) during binary fission. Extrinsic resistance arises via horizontal gene transfer (HGT), which allows acquisition of resistance genes from other organisms through three main processes: transformation (uptake of free DNA from the environment), transduction (bacteriophage-mediated gene transfer), and conjugation (direct plasmid transfer via pilus). Both intrinsic and extrinsic mechanisms collectively contribute to the persistence and spread of multidrug resistant phenotypes (Wang et al., 2023).

#### 3.2.2 Acquired resistance

Acquired resistance (Figure 2b) occurs when bacteria that were formerly susceptible to antibiotics evolve or acquire novel genetic characteristics that make them resilient to drug treatment. This can occur through two primary mechanisms: spontaneous mutations can modify antibiotic targets, metabolic processes, or membrane permeability. Vertical gene transfer, where DNA is transferred from parent to offspring during reproduction (usually by binary fission in bacteria), allows these mutations to persist within a lineage. More critically, horizontal gene transfer (HGT) whereby resistance genes are exchanged between bacteria, often across species has become a major driver of the global spread of resistance (Munita and Arias, 2016; Partridge et al., 2018; Naghavi et al., 2024). A prime example is *Mycobacterium tuberculosis*, which has developed resistance to rifampicin due to *rpoB* gene mutations that alter the RNA polymerase β subunit, rifampicin’s main molecular target. These mutations hamper the action of the drug, resulting in treatment failure in drug-resistant TB cases; tuberculosis remains one of the deadliest antibiotic-resistant diseases worldwide (Global Tuberculosis Report, 2024). Another instance is *Escherichia coli* acquiring extended-spectrum beta-lactamase (ESBL) genes such as blaCTX-M, which confer resistance to third-generation cephalosporins. These genes are usually plasmid-borne, making them easily transmissible between Gram-negative bacteria (Widodo et al., 2023; Sintondji et al., 2023). Likewise, *Staphylococcus aureus* can acquire the *mecA* gene via HGT, resulting in methicillin-resistant *S. aureus* (MRSA). The *mecA* gene encodes the penicillin-binding protein PBP2a, which has low affinity for β-lactams, allowing cell wall synthesis to proceed despite antibiotic pressure (Nguyen-Thi et al., 2024). *Klebsiella pneumoniae* has emerged as a major threat because it can acquire and disseminate carbapenemase genes such as KPC, NDM, or OXA-48, which produce enzymes that hydrolyze carbapenems, the last line of defense for many bacterial infections. These examples illustrate the remarkable genetic adaptability of bacterial populations exposed to antibiotic pressure and highlight the importance of global surveillance and stewardship (Naghavi et al., 2024).

##### 3.2.2.1 Mechanism of vertical gene transfer

Vertical gene transfer in bacteria occurs when a parent cell divides and passes its DNA to its offspring (Baharoglu and Mazel, 2014). The process begins with DNA replication, during which the bacterial chromosome is duplicated; the cell then grows and divides through binary fission, producing two identical daughter cells (Holmes et al., 2016). Each daughter cell inherits a full copy of the genetic material from its parent generation. If the parent cell harbors genes conferring antibiotic resistance, these genes are automatically transmitted, ensuring that resistance persists in the population across generations even in the absence of new external resistance inputs (Chen et al., 2023) (Figure 2).

##### 3.2.2.2 Mechanisms of horizontal transfer

Although mutations and selective pressure have been the core drivers of antibiotic resistance evolution for decades, the contribution of horizontal gene transfer (HGT) is often downplayed in public discourse, yet it remains one of the most effective means by which resistance traits spread rapidly within and between bacterial communities (Munita and Arias, 2016). In contrast to vertical transmission, where parent bacteria transfer genetic information directly to their offspring, HGT allows bacteria to exchange genetic material horizontally, even across species or genera. This process can produce sudden and dramatic shifts in a bacterium’s capabilities where the rapid acquisition of antibiotic resistance genes to which the organism previously had no exposure (Brockhurst et al., 2019). Three main pathways underpin HGT: transformation, where bacteria absorb free DNA fragments from their surroundings (often from lysed cells); transduction, where bacteriophages shuttle genetic material between hosts; and conjugation, which involves direct plasmid exchange through cell-to-cell contact using a pilus (Karkman et al., 2018; Chen et al., 2023). These pathways are especially potent in environments such as wastewater treatment plants, hospital settings, and agricultural soils contaminated by pharmaceutical runoff, all of which sustain high microbial diversity and selective pressure (Alem et al., 2025). Among these, plasmid-mediated conjugation poses a particular threat. Plasmids frequently carry clusters of resistance genes sometimes bundled with genes that confer tolerance to heavy metals, disinfectants, or biocides enabling the simultaneous spread of multi-drug resistance and other survival traits when plasmids move between microbes (Castaneda-Barba et al., 2024). The consequences become even more serious when HGT bridges environmental microbes and human pathogens. In these scenarios, benign environmental bacteria act as genetic “middlemen,” channeling resistance genes into clinically relevant strains (Gillings et al., 2017; Berglund, 2015). This gene flow turns natural reservoirs such as rivers, soil, and wastewater into breeding grounds for emerging resistance threats, especially where contamination by hospital or pharmaceutical waste accelerates the cycle (Karkman et al., 2018; La Rosa et al., 2025) (Figure 2).

### 3.3 The role of biofilms in antibiotic resistance

In the microbial environment, survival is often contingent on communal interactions. Biofilms represent a sophisticated adaptive strategy that enables bacteria to persist under hostile conditions (Flemming and Wingender, 2010; Dykes et al., 2026). They occur on medical devices, dental plaque, and environmental surfaces, forming complex, structured communities of microorganisms embedded in a self-produced extracellular polymeric matrix (EPM). Antibiotics have poor penetration through established biofilms, while cells in deep layers of biofilms enter low-active or quasi-dormant states, thus reducing sensitivity to antibiotics targeted at actively reproducing bacteria (Dehestaniathar et al., 2021; Wang et al., 2024; Liu et al., 2023). Additionally, biofilms play an important role as genetic information exchange centers; the high-density nature of the bacterial population promotes gene transmission via plasmids, transposons, and integrons (Hall and Mah, 2017; Castaneda-Barba et al., 2024). Resistance traits can be spread among the biofilm from a single resistant cell, leading to increased survival of the biofilm community as a whole. Biofilm infections therefore pose significant treatment issues and tend to be chronic. Diseases like sinusitis, endocarditis, cystic fibrosis lung infections, and device-related infections, including those due to urinary catheters, joint prostheses, and heart valves, are all greatly affected by the presence of biofilms. In clinical settings, colonization of surfaces and equipment acts as sources of multidrug-resistant bacteria, indicating that bacterial persistence does not only come about as a result of genetic changes but is additionally due to the protective niche provided by biofilms. In addition to being involved in antibiotic resistance by virtue of their structure, biofilms also act as signaling networks that control the process of spreading resistance genes. Autoinducers are small signaling molecules, such as AHLs for Gram-negative bacteria and AI-2 for Gram-positive and Gram-negative bacteria, which make up the core components of the quorum sensing signaling pathway. Through this signaling system, bacteria are able to sense when the population reaches the threshold number needed to activate certain group functions, including biofilm formation, development of pathogenicity, and most importantly, horizontal gene transfer (HGT). For example, *P. aeruginosa* uses QS to enhance its ability to engage in conjugation for transferring carbapenemase genes like *bla*KPC via plasmids between neighboring bacteria (Madsen et al., 2012; Lamba et al., 2018; Qiu et al., 2024). Several studies demonstrate that antibiotic concentrations below the lethal range can disrupt the regulatory mechanisms in microbes. According to modern research, low levels of ciprofloxacin, for instance, 0.1 μg/L, which is often found in wastewater, may act as quorum-sensing chemicals, thus stimulating the secretion of biofilm matrix and speeding up the exchange of antibiotic resistance genes (Gupta et al., 2016). In such a way, there occurs a vicious circle when substances that are supposed to kill microorganisms actually stimulate them to exhibit cooperative behavior and develop resistance. The presence of biofilm creates a situation where such substances become the genetic reservoirs, the “supermarket” of antibiotic resistance genes, such as *bla*NDM-1 (Zhao et al., 2020; Wang, et al., 2023).

### 3.4 Biochemical mechanisms of antibiotic resistance in bacteria

Beyond the structural and genetic adaptations that enable bacteria to acquire and transmit resistance, the actual survival of a resistant bacterium in the presence of an antibiotic ultimately depends on its biochemical arsenal. Bacteria have evolved a remarkably diverse set of molecular strategies to neutralize, evade, or circumvent the lethal action of antimicrobial agents. These biochemical mechanisms result in enzymatic inactivation of the drug, modification of its molecular target, active expulsion via efflux pumps, restriction of cellular entry, and metabolic rerouting which rarely operate in isolation. In clinical and environmental MDR strains, multiple mechanisms frequently co-occur on the same organism, compounding treatment failure and rendering even last-resort antibiotics ineffective (Blair et al., 2015; Munita and Arias, 2016) (Figure 3, Table 1).

**FIGURE 3:**
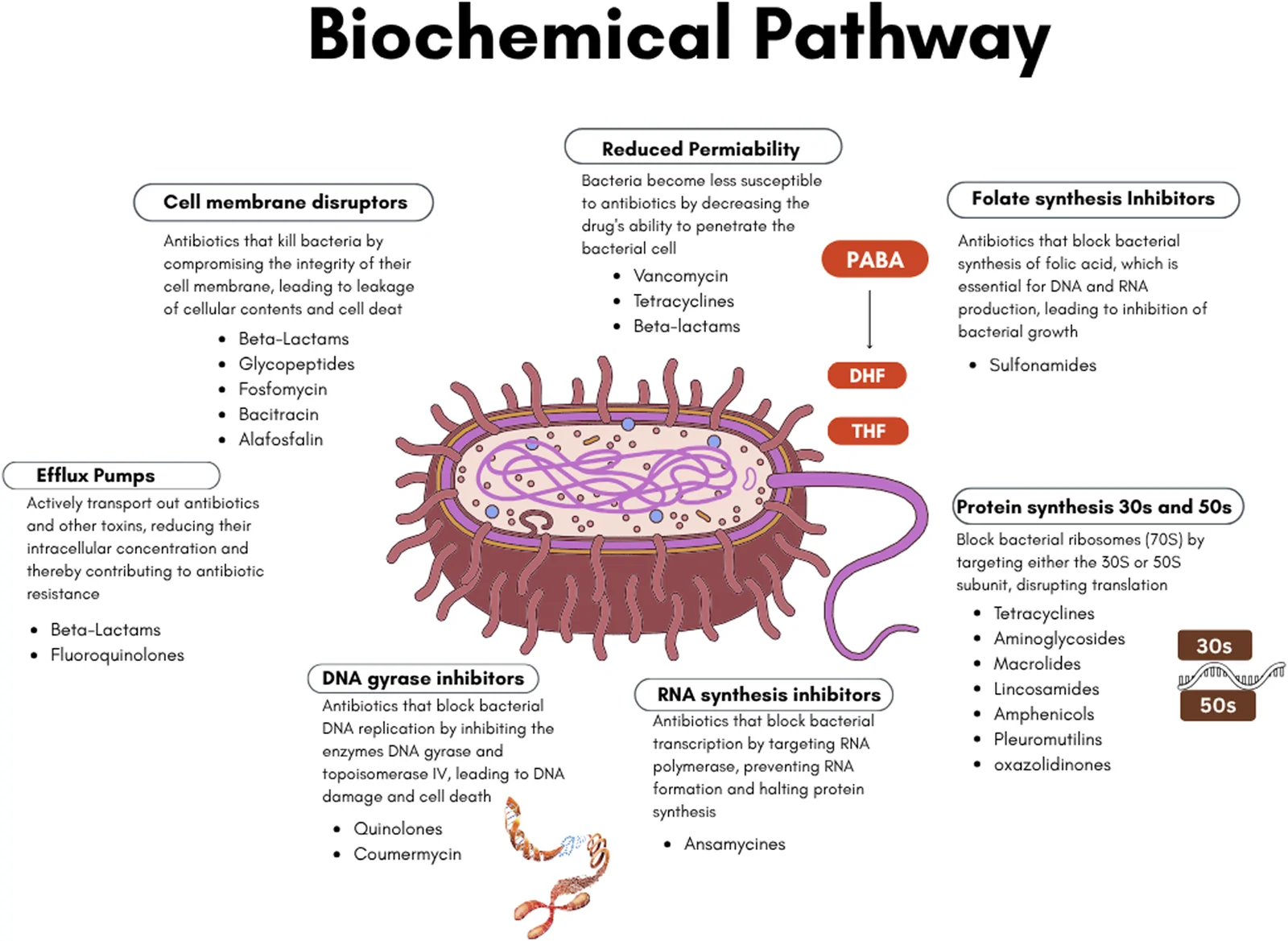
Biochemical pathways targeted by antibiotics in bacterial cells. The figure highlights the key cellular processes where different classes of antibiotics exert their effects. Antibiotics act at various stages, including cell wall synthesis (e.g., Penicillins, Vancomycin), protein synthesis (e.g., Tetracyclines, Macrolides, Aminoglycosides), nucleic acid synthesis (e.g., Fluoroquinolones, Rifamycins), and folate synthesis (e.g., Sulfonamides, Trimethoprim). The diagram also depicts the interaction of antibiotics with specific bacterial enzymes or structures, inhibiting critical processes necessary for bacterial growth and survival (Keller and Dörr, 2023; Syroegin et al., 2022).

**TABLE 1:** Overview of major antibiotic classes, mechanisms, resistance genes, and clinical applications.

Antibiotic class	Mechanism of action	Key resistance genes	Typical diseases treated	References
Beta-lactams (penicillins, cephalosporins, carbapenems)	Inhibit bacterial cell wall synthesis by binding to penicillin-binding proteins (PBPs)	*blaTEM, blaSHV, blaCTX-M, mecA, NDM-1*	Pneumonia, urinary tract infections (UTIs), skin infections, sepsis, meningitis	Bush and Bradford (2016), Munita and Arias (2016)
Aminoglycosides (e.g., gentamicin, amikacin)	Inhibit protein synthesis by binding to the 30S ribosomal subunit, causing misreading of mRNA	*aac(6′)-ib, aph(3′), ant(2″), rmtB*	Septicemia, endocarditis, complicated UTIs, tuberculosis	Ramirez and Tolmasky (2010), Garneau-Tsodikova and Labby (2016)
Fluoroquinolones (e.g., ciprofloxacin, levofloxacin)	Inhibit DNA gyrase and topoisomerase IV, halting DNA replication	*qnrA, qnrB, gyrA* and *parC* mutations, *aac(6′)-Ib-cr*	Respiratory tract infections, gastrointestinal infections, UTIs, prostatitis	Robicsek et al. (2006), Hooper and Jacoby (2016)
Macrolides (e.g., erythromycin, azithromycin)	Block protein synthesis by binding to the 50S ribosomal subunit	*erm(B), mef(A), msr(D)*	Respiratory infections (e.g., bronchitis, pneumonia), STDs, skin infections	Leclercq (2002), Dinos (2017)
Tetracyclines (e.g., doxycycline, tetracycline)	Bind to the 30S ribosomal subunit to inhibit tRNA binding and prevent protein elongation	*tet(A), tet(M), tet(K)*	Acne, lyme disease, respiratory infections, STDs, malaria	Chopra and Roberts (2001), Grossman (2016)
Glycopeptides (e.g., vancomycin)	Inhibit cell wall synthesis by binding to D-Ala-D-Ala terminus of peptidoglycan precursors	*vanA, vanB*	MRSA infections, endocarditis, *Clostridium* difficile-associated diarrhea	Stogios and Savchenko (2020)
Sulfonamides (e.g., sulfamethoxazole)	Inhibit folic acid synthesis by targeting dihydropteroate synthase	*sul1, sul2, sul3*	UTIs, pneumocystis pneumonia (in HIV patients), ear infections	Sköld (2000), Venkatesan et al. (2023)
Oxazolidinones (e.g., linezolid)	Bind to 50S ribosomal subunit and prevent formation of the initiation complex for protein synthesis	*cfr, optrA*	MRSA, VRE (vancomycin-resistant *Enterococci*), pneumonia	Chen et al. (2019), Gillieatt and Coleman (2024)
Polymyxins (e.g., colistin, polymyxin B)	Disrupt bacterial outer membrane by binding to lipopolysaccharides	*mcr-1, mcr-2*	Multidrug-resistant gram-negative infections, particularly in ICU settings	Liu et al. (2016)
Rifamycins (e.g., rifampicin)	Inhibit bacterial RNA polymerase and block transcription	*rpoB* mutations	Tuberculosis, leprosy, meningococcal prophylaxis	Campbell et al. (2001), Mosaei and Harbottle (2019)
Lipopeptides (e.g., daptomycin)	Insert into bacterial membrane and cause depolarization, leading to cell death	Resistance due to mutations in *mprF, liaFSR*	Complicated skin infections, bacteremia, right-sided endocarditis	Tran et al. (2015), Miller et al. (2016)

#### 3.4.1 Enzymatic degradation or modification

One of the most direct ways bacteria defend themselves against antibiotics is by producing enzymes that chemically dismantle the drug before it can cause harm (Bush and Bradford, 2016). A classic example is the production of β-lactamases, which break open the β-lactam ring found in penicillins, cephalosporins, and related antibiotics (Munita and Arias, 2016). Once that ring is compromised, the antibiotic loses its activity and becomes therapeutically useless (Nikaido, 2009). A well-known group is the Extended Spectrum β-Lactamases (ESBLs), for example, CTX-M enzymes, which are commonly found in *E. coli* and *K. pneumoniae* (Widodo et al., 2023). These enzymes allow these bacteria to resist third-generation cephalosporins that are widely used in hospitals. Even more concerning are the carbapenemases, such as NDM-1 (New Delhi Metallo-β-lactamase), which can deactivate carbapenems, antibiotics often considered our last line of defense against multidrug-resistant bacteria. These enzymes are frequently carried on plasmids, which makes it easy for resistance to spread rapidly through bacterial populations by horizontal gene transfer (Naghavi et al., 2024; Pal et al., 2015) (Figure 3, Table 1).

#### 3.4.2 Target modification

Some antibiotics work by binding to specific bacterial proteins or structures, effectively shutting down vital cellular functions (Davies and Davies, 2010). In response, bacteria can modify these target sites, preventing the drug from attaching properly (Chen et al., 2023). A well-known example is methicillin-resistant *S. aureus* (MRSA), which carries the *mecA* gene. This gene encodes a modified penicillin-binding protein (PBP2a) that has a low affinity for β-lactam antibiotics, allowing the bacteria to continue synthesizing their cell walls even in the presence of these drugs (Nguyen-Thi et al., 2024). Similarly, *M. tuberculosis* can develop mutations in its *rpoB* gene, which encodes the β subunit of RNA polymerase rifampicin’s primary target. These mutations change the drug’s binding site, dramatically reducing its effectiveness and complicating tuberculosis treatment (Global Tuberculosis Report, 2024; Keller and Dörr, 2023) (Figure 3, Table 1).

#### 3.4.3 Efflux pumps

Efflux pumps actively expel antibiotics from the cell before these drugs can accumulate to levels that would actually kill or inhibit the bacteria (Li et al., 2015; Blair et al., 2015). What makes these pumps particularly troublesome is that many are nonspecific, meaning a single pump can expel multiple classes of antibiotics (Chen et al., 2023). For example, *P. aeruginosa* relies heavily on the MexAB-OprM efflux system, which contributes to its resistance to β-lactams, fluoroquinolones, and chloramphenicol (Lister et al., 2009; Mazza et al., 2025). Similarly, *E. coli* deploys the *AcrAB*-TolC efflux system to push out tetracyclines and various other antibiotics (Gaurav et al., 2023). A major challenge is that bacteria can upregulate these pumps in response to environmental stressors like sub-lethal antibiotic concentrations. This adaptive response allows them to boost their activity under pressure, making treatment failures more likely. Any in-depth knowledge of the efflux-mediated resistance process would have to take into account some of the key families of transporters involved in the process, especially in environmental and medical contexts. The RND family is one of the most medically and environmentally important groups: The tripartite RND systems like the MexAB-OprM in *P. aeruginosa* and the *AcrAB*-TolC in *E. coli* are present in the inner membrane, periplasm, and outer membrane, and therefore allow for active efflux of a broad range of structurally different compounds, which include β-lactams, fluoroquinolones, tetracyclines, and chloramphenicol (Blair et al., 2015; Hooper and Jacoby, 2016; Klemm et al., 2018). It is crucial to highlight that the RND pump family provides both intrinsic and acquired antibiotic resistance in the Gram-negative environmental bacteria and can be regulated in conjunction with heavy metal resistance operons and become a part of co-selection pressures due to antibiotics and heavy metals in polluted aquatic ecosystems (Seiler and Berendonk, 2012; Pal et al., 2017). Another significant family is the ATP-binding cassette (ABC) transporters used primarily by Gram-positive pathogens, such as *S. aureus*, and environmental actinobacteria. These transporters use the energy of hydrolysis of ATP to drive the efflux of a wide array of antibiotics, such as macrolides and streptogramins, and are used intrinsically by resistomes present in the soil prior to clinical antibiotics’ use (D'Costa et al., 2011; Leclercq, 2002). MATE transporters have been identified in both clinical isolates and environmental samples, including wastewater treatment effluents and river sediments, and have been implicated in fluoroquinolone resistance in non-pathogenic bacteria under sub-inhibitory selective pressure. Collectively, the substrate promiscuity, structural diversity, and wide environmental distribution of RND, ABC, and MATE transporter families explain why efflux-mediated resistance is so difficult to contain, a single resistant cell may simultaneously harbour pumps from multiple superfamilies, each targeting a distinct antibiotic class (Gupta et al., 2016; Zhao et al., 2020) (Figure 3, Table 1).

#### 3.4.4 Reduced permeability

Before antibiotics can do their job inside a bacterial cell, they first need to cross the bacterial membrane barriers. Some bacteria have evolved to reduce membrane permeability, either by altering membrane structure or by decreasing the production of porins, membrane proteins that act like channels for antibiotic entry. A well-documented example is *K. pneumoniae*, which can downregulate or mutate OmpK35 and OmpK36 porins. When these porins are under-expressed, the entry of β-lactam antibiotics, including carbapenems, is restricted (Chen et al., 2023). This effect is even more serious when combined with β-lactamase production, creating bacteria with very high levels of resistance. In general, Gram-negative bacteria are harder to penetrate than Gram-positive ones because they naturally have an extra outer membrane that blocks many antibiotic molecules. When this barrier is further strengthened by porin loss or mutation, even our strongest antibiotics can become ineffective (Nikaido, 2009) (Figure 3, Table 1).

#### 3.4.5 Metabolic bypass

Some antibiotics work by disrupting critical metabolic pathways, such as the synthesis of folic acid, which is essential for nucleic acid production (Munita and Arias, 2016). In response, bacteria may evolve alternative biochemical routes or acquire enzymes that let them bypass the blocked step. For example, *Streptococcus pneumoniae* and *Haemophilus influenzae* can develop resistance to trimethoprim-sulfamethoxazole, a drug combination that targets folate synthesis. These bacteria overcome the block by overproducing dihydrofolate reductase or by acquiring a resistant variant of the gene that encodes it (Tiseo et al., 2020; Syroegin et al., 2022). This workaround allows bacterial growth to continue even in the drug’s presence. A similar bypass happens with some strains of *E. faecalis*, which have shown resistance to linezolid, an antibiotic that binds the 50S ribosomal subunit to stop protein synthesis. These strains evade linezolid by producing altered ribosomal RNA or ribosomal proteins that reduce the antibiotic’s binding affinity, enabling protein synthesis to proceed and the bacteria to survive (Halawa et al., 2024) (Figure 3; Table 1).

### 3.5 Influence of metals on antibiotic resistance development

Antibiotic resistance is not merely a result of overusing antibiotics; environmental influences, especially pollutants like heavy metals, significantly contribute to unexpected bacterial evolution (Seiler and Berendonk, 2012). Co-selection is a vital yet frequently overlooked process in the story of antibiotic resistance, where non-antibiotic substances particularly metals such as zinc (Zn^2+^), copper (Cu^2+^), mercury (Hg^2+^), cadmium (Cd^2+^), and arsenic (As^3+^) indirectly promote antibiotic resistance by impacting similar survival strategies (Gillieatt and Coleman, 2024; Wang et al., 2023).

#### 3.5.1 Shared genetic platforms

Resistance genes for metals and antibiotics often coexist on the same mobile genetic elements including plasmids, integrons, and transposons. For instance, a single plasmid may encode both a copper-resistance operon (cop) and antibiotic-resistance genes (blaCTX-M, qnr, tet) (Xu and Lin, 2024). Exposure to metals such as copper 1 or zinc activates these operons, inadvertently maintaining or enhancing antibiotic resistance even in the absence of antibiotics (Pal et al., 2017). This co-resistance links unrelated stressors through physical gene proximity, ensuring that environmental pollution indirectly sustains antimicrobial resistance (Seiler and Berendonk, 2012; Dickinson et al., 2019).

#### 3.5.2 Mechanism of antibiotic resistance driven by heavy metals

Metals not only select for co-carried genes but also trigger bacterial defense systems that overlap with antibiotic resistance pathways (Seiler and Berendonk, 2012; Vats et al., 2022). For example, zinc (Zn^2+^) boosts the expression of beta-lactamase genes, such as blaTEM, which break down penicillins (Xu and Lin, 2024). Copper (Cu^2+^) and mercury (Hg^2+^) can initiate stress responses like the SOS system, leading to higher mutation rates and mobilization of resistance elements (Gillings et al., 2015). These stress-triggered shifts can cause DNA rearrangements, producing more adaptable bacterial variants (Wang et al., 2023). Cadmium (Cd^2+^) can induce multidrug efflux pumps like *tetA* and acrAB, increasing antibiotic tolerance, while arsenic (As^3+^) through the *ars* operon is often co-localized with erm and bla genes, reinforcing co-selection in contaminated environments (Pal et al., 2017; Dickinson et al., 2019) (Table 2).

##### 3.5.2.1 Zinc (Zn^2+^)

Zinc acts as a potent co-selective agent for antibiotic resistance by fostering conditions where resistance genes can thrive (Gillieatt and Coleman, 2024). Exposure to zinc has been linked to the upregulation of bla genes (beta-lactamases) and aminoglycoside resistance determinants (Vats et al., 2022; Ramirez and Tolmasky, 2010). These genes are often found near metal resistance operons like czc (cadmium, zinc, cobalt), which provides resistance to zinc, cobalt, and cadmium. This physical linkage allows co-selection to occur even without antibiotics present, making zinc-contaminated runoff and untreated sewage key reservoirs for resistant bacteria (Xu and Lin, 2024). By boosting beta-lactamase production, zinc also enhances horizontal gene transfer potential. Notably, zinc concentrations as low as 0.1–0.5 mg/L in soil porewater have been shown to maintain co-selective pressure on *bla* and aminoglycoside resistance genes in the absence of any antibiotic exposure (Pal et al., 2017). Zinc-based veterinary feed additives represent a major agricultural source of this selective pressure; while the EU prohibited their prophylactic use in 2022, they remain widely used across Asia and Latin America, sustaining zinc-driven co-selection in livestock-associated environments globally (Xu and Lin, 2024; Garneau-Tsodikova and Labby, 2016) (Table 2).

##### 3.5.2.2 Copper (Cu^2+^)

Copper contributes to the spread of antimicrobial resistance by promoting gene mobilization and raising bacterial mutation rates. Resistance genes for beta-lactams and fluoroquinolones, such as bla and qnr, frequently sit on plasmids and integrons that also carry copper resistance genes like the cop operon (Wang, et al., 2023). This co-localization enables bacteria to gain simultaneous resistance under copper exposure. Industrial runoff and mining waste create copper-rich environments that exert selective pressure, enhancing the spread of multidrug-resistant strains. Critically, the *pco* and *cop* copper resistance operons are frequently co-located on IncHI2-type plasmids that also carry *blaCTX-M* extended-spectrum beta-lactamase genes, meaning copper tolerance and ESBL production are physically linked and co-selected as a single genetic unit (Pal et al., 2017; Gillings et al., 2015). The widespread application of copper-rich pig slurry to agricultural soils has been directly associated with ARG enrichment in soil microbiomes, establishing farmland as a significant reservoir for copper-driven multi-drug resistance beyond industrial settings (Wang, et al., 2023) (Table 2).

##### 3.5.2.3 Mercury (Hg^2+^)

Mercury is a strong driver of antibiotic resistance because it can activate the bacterial SOS response, increasing mutation rates. Resistance genes like bla (beta-lactams) and tet (tetracyclines) often occur alongside mercury resistance genes within the mer operon, which detoxifies mercury while co-selecting for antibiotic resistance (Pal et al., 2017). Hospitals and industrial discharges are major mercury sources, creating environments where mercury and resistance traits flourish together. The *mer* operon’s association with the Tn*21* transposon family is particularly significant: this mobile element contains an integron platform capable of capturing additional antibiotic resistance gene cassettes, explaining why mercury-contaminated environments consistently yield multi-resistant rather than single-resistant phenotypes (Dickinson et al., 2019; Gillings et al., 2015). Beyond industrial discharges, dental amalgam waste and chlor-alkali plant effluents represent underappreciated mercury sources that create clinically proximate environments where *mer*-linked resistance can transfer directly into healthcare-associated microbiomes (Vats et al., 2022) (Table 2).

##### 3.5.2.4 Arsenic (As^3+^)

Arsenic exposure is strongly linked to co-regulation of multiple antibiotic resistance genes. The ars operon, which confers arsenic resistance, is often found near erm genes (macrolide resistance) and bla genes (Vats et al., 2022). This genetic clustering helps co-select for antibiotic resistance in arsenic-polluted settings. Arsenic-laden industrial effluents and contaminated water bodies are therefore high-risk sites for multidrug-resistant bacterial populations. Metagenomic analyses of pristine soils predating the antibiotic era have identified *arsB* and *arsC* gene variants, confirming the *ars* operon as an ancient environmental resistance determinant now being co-opted under modern selective pressure rather than *de novo* selected by antibiotic use (Dickinson et al., 2019). Groundwater arsenic contamination across South Asia particularly in Bangladesh and West Bengal, where co-occurring antibiotic residues from pharmaceutical effluents create compounding selective pressure represents a high-priority context for monitoring arsenic-driven ARG co-selection in drinking water sources Xu and Lin, 2024) (Table 2).

##### 3.5.2.5 Cadmium (Cd^2+^)

Cadmium promotes co-selection by activating efflux pump genes like *tetA* and *acrAB*, which pump out tetracyclines and other drugs. It also triggers expression of cadmium-specific genes like cadA, strengthening overall metal and antibiotic resilience. This dual action boosts gene transfers and helps resistant strains persist in cadmium-contaminated soil, agricultural runoff, or mining sites. It is important to distinguish this mechanism as cross-induction rather than co-resistance: cadmium exposure transcriptionally upregulates efflux pumps particularly *tetA* and *acrAB* whose primary substrates are antibiotics rather than the metal itself, meaning antibiotic efflux capacity is enhanced even in the complete absence of antibiotic exposure or shared genetic platforms (Pal et al., 2017; Vats et al., 2022). This mechanistically distinct pathway makes cadmium particularly insidious as a co-selective agent, as standard co-resistance screening focused on shared plasmid architecture will systematically under detect cadmium-driven resistance enrichment in contaminated soils and agricultural runoff (Venkatesan et al., 2023; Gillieatt and Coleman, 2024). Class 1 integrons have particular structural importance, which function as natural gene-capture platforms in Gram-negative bacteria and can simultaneously acquire both metal and antibiotic resistance gene cassettes at their attI recombination site (Grossman, 2016; Gillings et al., 2015). This integron-mediated co-assembly rather than simple co-location is the primary molecular mechanism by which multi-resistance is rapidly constructed and disseminated across species boundaries in metal-contaminated environments, making integron prevalence a meaningful proxy indicator for co-selection risk in environmental surveillance (Pal et al., 2017) (Table 2).

#### 3.5.3 Role of biofilms in metal-driven resistance

Biofilms are the ideal training ground for resistance to thrive in metal-rich environments such as hospital sinks, aquaculture ponds, or polluted soils (Harrison et al., 2007). Within these dense microbial communities, bacteria form protective layers of extracellular polymeric substances, which trap nutrients and pollutants alike. Within these metal-exposed communities, a critical persistence-then-acquisition dynamic operates: phenotypically tolerant subpopulations lacking heritable resistance genes survive metal stress through physiological adaptations, remaining viable long enough to acquire resistance determinants from neighboring cells via HGT (Flemming and Wingender, 2010; Madsen et al., 2012). This two-stage process, tolerance providing survival time, HGT providing heritable resistance, means that biofilms in metal-contaminated settings are disproportionately effective as ARG dissemination hotspots compared to planktonic environments, where cells lacking resistance genes are eliminated before gene transfer can occur (Balta et al., 2025). This sticky matrix also acts as a reservoir, accumulating metals like copper, zinc, and cadmium, which increases selective pressure for bacteria to maintain survival traits (Flemming and Wingender, 2010). Inside these micro-ecosystems, close cell-to-cell contact accelerates genetic exchange through horizontal gene transfer, allowing bacteria to swap resistance genes rapidly (Zhang et al., 2018; Reichert et al., 2021). The biofilm’s physical barrier not only shields bacteria from external threats but also creates localized hotspots where stress-induced systems stay active including the SOS response and efflux pump activation pushing bacteria toward greater tolerance for both metals and antibiotics (Madsen et al., 2012). As a result, biofilms serve as incubators where resistance can proliferate and diversify, sustaining reservoirs of multi-resistant pathogens in medical and environmental settings (Salviano et al., 2026; Stachurska et al., 2020).

**TABLE 2:** Heavy metal-driven co-selection of antibiotic resistance.

Metal	Co-selected antibiotics	Targeted resistance genes/mechanisms	Effect on resistance	Environmental sources	References
Zinc (Zn^2+^)	Beta-lactams, aminoglycosides	Genes encoding beta-lactamases (e.g., *bla* genes), aminoglycoside resistance genes; linked with metal resistance operons (e.g., *czc* operon)	Upregulates beta-lactamase production	Agricultural runoff, sewage	Seiler and Berendonk (2012), Vats et al. (2022)
Copper (Cu^2+^)	Beta-lactams, fluoroquinolones	Mobilizable genetic elements (plasmids, integrons) carrying bla, qnr (fluoroquinolone resistance), and copper resistance genes (*cop* operon)	Induces mutation rates, enhances gene mobilization	Industrial waste, mining	Seiler and Berendonk (2012), Vats et al. (2022), Gillings et al. (2015) , Pal et al. (2015)
Mercury (Hg^2+^)	Beta-lactams, tetracyclines	Resistance genes linked to mercury resistance operon *mer* and bla beta-lactamase genes; tetracycline resistance genes (e.g., *tet* genes)	Activates SOS response, increases mutation rates	Hospital waste, industrial discharges	Seiler and Berendonk (2012), Vats et al. (2022)
Arsenic (As^3+^)	Macrolides, beta-lactams	Co-regulation of arsenic resistance genes (*ars* operon) with erm (macrolide resistance), bla genes	Induces expression of multiple resistance genes	Contaminated water, industrial waste	Seiler and Berendonk (2012), Vats et al. (2022), Dickinson et al. (2019)
Cadmium (Cd^2+^)	Tetracyclines, aminoglycosides	Efflux pump genes (e.g., tetA, acrAB), metal resistance genes like *cadA*, and aminoglycoside resistance genes	Increases efflux pump activity and resistance gene mobilization	Agricultural runoff, mining	Seiler and Berendonk (2012), Vats et al. (2022), Pal et al. (2017), Pal et al. (2015)

#### 3.5.4 Comparative effect of heavy metals and antibiotic resistant genes as key drivers

The role of heavy metals in promoting antibiotic resistance extends far beyond mere co-contamination (Seiler and Berendonk, 2012). Metals like copper (Cu), zinc (Zn), and mercury (Hg) persist in soils and sediments for decades, creating chronic selective pressure that favors bacteria with dual resistance mechanisms (Pal et al., 2017). For instance, the czcA efflux pump in *P. aeruginosa* can simultaneously expel zinc, cadmium, and β-lactam antibiotics, effectively linking metal tolerance with carbapenem resistance. This co-selection is remarkably efficient: heavy metal pollution has been shown to increase plasmid conjugation rates, boosting the spread of *bla*NDM-1 and related resistance elements (Zhang, et al., 2018; Wang, et al., 2023). These dynamics transform polluted aquatic ecosystems into “training grounds” where bacteria evolve cross-resistance, an issue worsened by industrial discharges that often exceed regulatory safety thresholds (Global Antibiotic Resistance Surveillance Report, 2025). In contrast, antibiotic resistance genes (ARGs) function as “ready-made weapons” for bacteria, with their mobility largely determining the speed and scale of spread (Balta et al., 2025). Plasmids like IncF, which frequently carry blaCTX-M-15, and class 1 integrons like sul1, disseminate rapidly through urban wastewater biofilms, especially where sub-inhibitory antibiotic residues such as 500 ng/L ciprofloxacin provide direct selective pressure. In pharmaceutical effluent streams, *bla*NDM-1 prevalence has been found to correlate more strongly with unmetabolized antibiotics than with metal levels, suggesting ARGs remain the primary drivers of clinical resistance when drug residues are abundant (Stachurska et al., 2020; Venkatesan et al., 2023). Yet these factors synergize in practice: aquaculture farms that use copper-contaminated feed show *mcr-1* (colistin resistance) levels up to eight times higher than control sites, demonstrating how metals can stabilize ARGs in the food chain and wider environment (Wang et al., 2023) (Table 2).

## 4 Multi-drug-resistant bacteria (MDR)

The global rise in antibiotic-resistant bacteria has reached critical levels, largely driven by the misuse of antibiotics in clinical and agricultural practices. This trend has led to the emergence of multidrug resistance (MDR) strains that no longer respond to several antibiotic classes (Murray et al., 2022). MDR arises when bacteria acquire mechanisms conferring resistance to multiple antibiotic classes, substantially complicating treatment and threatening the effectiveness of modern medicine (Magiorakos et al., 2012; Ventola, 2015). Bacterial resistance develops through evolutionary adaptations that enable pathogens to survive exposure to antimicrobial agents designed to eliminate them (Blair et al., 2015; MacLean and San Millan, 2019). This evolutionary process is accelerated by two major forces: antibiotic misuse in healthcare and livestock production and environmental selection pressures arising from pharmaceutical and industrial waste contamination (Klein et al., 2024; Global Antibiotic Resistance Surveillance Report, 2025). When exposed to sublethal antibiotic concentrations, bacteria activate resistance mechanisms such as enzymatic inactivation (e.g., β-lactamases), target site modification, or efflux pumps, rendering drugs ineffective. These traits are readily disseminated through HGT with plasmids and integrons acting as mobile genetic elements (Nikaido, 2009). The convergence of multiple resistance pathways gives rise to formidable MDR pathogens, particularly the ESKAPE group (*Enterococcus faecium, S. aureus, K. pneumoniae, A. baumannii, P. aeruginosa*, and *Enterobacter* spp.), which are responsible for most untreatable hospital-acquired infections (Pendleton et al., 2013; European Centre for Disease Prevention and Control, 2023). The COVID-19 pandemic further amplified this issue, with ICUs reporting concurrent outbreaks of carbapenem-resistant *A. baumannii* and *Candida auris*, demonstrating how global health crises can exacerbate antimicrobial resistance (Singh and Pandey, 2024; WHO, 2024). Environmental antibiotic contamination also plays a decisive role. Up to 90% of orally administered antibiotics are excreted unmetabolized and enter wastewater systems (Gothwal and Shashidhar, 2015). Inadequate treatment allows these residues to persist in aquatic and soil environments, sustaining reservoirs of resistance genes (Kafaiya et al., 2026). Prolonged exposure and cumulative selective pressures facilitate the transition from MDR to extensively drug-resistant (XDR) and ultimately pandrug resistance (PDR) forms, as defined by Magiorakos et al. (2012) and the World Health Organization (Table 3).

**TABLE 3:** Standardized classification of multidrug resistance (MDR), extensively drug-resistant (XDR), and pandrug-resistant (PDR) bacteria.

Resistance category	Definition	Clinical implication	Examples of pathogens	Prevalence and trends	Threat levels	References
MDR (multidrug resistance	Non-susceptibility to at least one agent in ≥3 antimicrobial categories	First-line treatments become ineffective; requires use of second-line or more toxic drugs	*Mycobacterium tuberculosis, E. coli, K. pneumoniae*	In the EU/EEA, third-generation cephalosporin-resistant *E. coli* bloodstream infections had an incidence of 10.35 per 100,000 populations in 2023. In India, *K. pneumoniae* showed a decline in susceptibility to imipenem from 58.5% to 35.6% between 2017 and 2023	High	ICMR (2024), Global Tuberculosis Report (2024) , Magiorakos et al. (2012) , Murray et al. (2022)
XDR (extensively drug-resistant)	Non-susceptibility to at least one agent in all but ≤2 antimicrobial categories (i.e., bacteria remain susceptible to only one or two categories	Limited treatment options; higher mortality risk	*Pseudomonas aeruginosa, Acinetobacter baumannii*	In 2019, there were approximately 25,060 new XDR-TB cases globally, a 22.5% increase from 2010.In a Greek hospital (2016–2023), colistin resistance in *A. baumannii* increased from 8.4% to 49.3%	Very high	Global Tuberculosis Report (2024) , Lagadinou et al. (2024) , Kubin et al. (2025), Tacconelli et al. (2018), Karakonstantis et al. (2024)
PDR (pandrug resistance)	Non-susceptibility to all agents in all antimicrobial categories	No known effective treatment; outcomes are often fatal or require experimental therapies	Rare cases of *K. pneumoniae, A. baumannii*	PDR pathogens are rare but pose significant treatment challenges due to resistance to all available antibiotics. Infections caused by PDR bacteria are associated with high mortality rates and limited treatment options	Critical	Murray et al. (2022) , Tacconelli et al. (2018) , Terzi et al. (2025) , Zampaloni et al. (2024), Karakonstantis et al. (2024)

### 4.1 Rising MDR/XDR/PDR trends across key bacterial infections, highlighting therapeutic escalation

The rise of AMR has severely compromised the efficacy of first-line antibiotics, compelling a global shift toward second-line and last-resort therapies (WHO, 2025). For instance, a 6 year retrospective study at a tertiary care center in Romania documented a sharp decline in susceptibility among *K. pneumoniae* isolates, with pandrug resistance (PDR) rising from 7.63% in 2018 to 17.55% in 2023 and extensively drug-resistant (XDR) strains increasing from 22.88% to 39.81% (Tălăpan et al., 2026). Similarly, India has witnessed a disturbing increase in XDR *A. baumannii* in bloodstream infections, with resistance to carbapenems reaching 76.15% of isolates, effectively nullifying multiple therapeutic options (Indian Council of Medical Research, 2024; Terzi et al., 2025). These patterns indicate a collapse in first-line defenses, necessitating the use of highly toxic or less accessible second-line antibiotics. Recent ICU-based studies confirm this alarming trend. In Poland, XDR organisms were detected in 22.6% of healthcare-associated infections, with *A. baumannii* being the most dominant. A parallel investigation in Western Greece found that 69.3% of Gram-negative bloodstream isolates qualified as XDR, drastically limiting clinical management options (European Centre for Disease Prevention and Control, 2023). This shift towards XDR and PDR phenotypes has been compounded by the overuse of antibiotics during the COVID-19 pandemic, which intensified selective pressure in healthcare environments (Klein et al., 2024; Indian Council of Medical Research, 2024). The consequences of PDR strains are particularly dire, often leading to prolonged hospitalization, elevated healthcare costs, and mortality rates surpassing 50% in some ICUs (Coppola et al., 2022; Global Tuberculosis Report, 2024). With nearly all available antibiotic classes rendered ineffective, treatment of PDR infections hinges on experimental therapies or compassionate-use antibiotics (Lagadinou et al., 2024). This has led to renewed global investment in antibiotic development; for example, Roche’s new compound, zosurabalpin, is undergoing advanced trials and could become the first new antibiotic class in over five decades to target Gram-negative bacteria like *Acinetobacter* (Zampaloni et al., 2024; Global Antibiotic Resistance Surveillance Report, 2025) (Table 4).

**TABLE 4:** Progression of antibiotic resistance necessitating shift from first-line to lastresort therapies.

Disease/pathogen	First-line antibiotics	Second-line/alternatives	Third-line/last-resort	Resistance status	References
Tuberculosis (*Mycobacterium tuberculosis*)	Isoniazid, rifampin	Fluoroquinolones, ethionamide, cycloserine	Bedaquiline, linezolid, delamanid	Widespread MDR/XDR; >500,000 rifampin-resistant TB cases in 2022	Global Tuberculosis Report (2024)
UTIs (*E. coli*)	TMP-SMX, nitrofurantoin, amoxicillin	Cephalosporins, fluoroquinolones	Carbapenems	MDR *E. coli* widespread; carbapenem resistance rising	Murray et al. (2022)
Gonorrhea (*Neisseria gonorrhoeae*)	Ceftriaxone, azithromycin	Spectinomycin, gentamicin	None (PDR reported)	XDR/PDR strains in United Kingdom, Japan, China	WHO (2025), Tacconelli et al. (2018)
Typhoid (*Salmonella typhi*)	Ampicillin, TMP-SMX, chloramphenicol	Fluoroquinolones	Azithromycin, carbapenems	XDR typhoid epidemic in Pakistan; spreading in South Asia	Klemm et al. (2018), Global Tuberculosis Report (2024) , Larsson et al. (2018), Murray et al. (2022)
Pneumonia (*S. pneumoniae*)	Penicillin, amoxicillin	Macrolides, cephalosporins	Fluoroquinolones, linezolid	Macrolide resistance >30% in asia	Lynch and Zhanel (2010), Murray et al. (2022)
Hospital-acquired infections (*K. pneumoniae*, *A. baumannii*)	Cephalosporins, piperacillin-tazobactam	Carbapenems	Colistin, tigecycline	XDR and PDR strains common in ICU settings	Global Antibiotic Resistance Surveillance Report (2025) , Tacconelli et al. (2018) , Kubin et al. (2025) , Zampaloni et al. (2024) , Codjoe and Donkor (2017)
MRSA (*Staphylococcus aureus*)	Methicillin (historical), oxacillin	Vancomycin, linezolid	Daptomycin, ceftaroline	MRSA is MDR; VRSA reported in United States and India	Chambers and DeLeo (2009), Nguyen-Thi et al. (2024) , Miller et al. (2016)
Cholera (*Vibrio cholerae*)	Doxycycline, azithromycin	Ciprofloxacin	3rd gen cephalosporins	MDR strains observed in Yemen, Haiti	WHO (2024), Murray et al. (2022)
Shigellosis (*Shigella* *flexneri,* *Shigella* spp.)	TMP-SMX, ampicillin	Ciprofloxacin, azithromycin	Ceftriaxone, carbapenems	XDR strains found in asia, africa, and now the United Kingdom	Thorley et al. (2023), Murray et al. (2022), Charles et al. (2025)

### 4.2 Critical drivers of multidrug resistance

The standard narrative around MDR points to clinical overuse and agricultural mismanagement. Both are legitimate but they miss a third, less visible engine: the tons of antibiotic-laden waste that pharmaceutical manufacturers and healthcare facilities release into the environment every day. An estimated 100,000–200,000 tons of antibiotics enter aquatic systems annually, a substantial fraction of it excreted unmetabolized, establishing the kind of chronic, low-dose selective pressure that drives resistance far more insidiously than acute exposure (Kümmerer, 2009; Tran et al., 2015). The geography of contamination is well-documented. Ciprofloxacin levels in India’s Musi River exceed safe ecological thresholds by more than 1,000-fold and carbapenemase genes (blaNDM-1) appear in both clinical and environmental isolates from the same basin (Terzi et al., 2025). China’s Pearl River Delta shows elevated *mcr-1* (colistin resistance) gene abundance near pharmaceutical manufacturing zones (Zhu et al., 2013). European wastewater treatment plants, often assumed to be reliable barriers, still fail to eliminate up to 70% of resistant *E. coli* (Berendonk et al., 2015; Bengtsson-Palme et al., 2018). These are not isolated incidents. They are snapshots of a global pattern. What makes contaminated aquatic environments particularly dangerous is that they do not merely harbor resistance, they amplify it. Biofilms in wastewater treatment plants increase horizontal gene transfer rates by up to 100-fold, allowing resistance genes such as blaCTX-M-15 and *qnrS* to spread aggressively within microbial communities (Karkman et al., 2019; Zhang et al., 2019). Even sub-inhibitory antibiotic concentrations as low as 1 μg/L are sufficient to maintain selection pressure. Heavy metals such as copper and zinc which are commonly found in hospital effluents compound this by acting as co-selective agents on shared genetic platforms, sustaining resistance even in the complete absence of antibiotics (Pal et al., 2017). The link between environmental contamination and clinical outcomes is no longer speculative. Genomic surveillance has confirmed identical MDR plasmids in riverine *E. coli* and clinical *K. pneumoniae* isolates from the same watersheds. More striking, environmental detection of *bla*NDM-1 preceded hospital outbreaks by several years, the environment was warning us before clinicians noticed (Walsh et al., 2011). The governance failure underlying this is stark. Only 12% of pharmaceutical manufacturers globally test their effluents for resistance genes. Roughly 45% of unused antibiotics are improperly discarded by households and healthcare facilities. No binding international discharge standards exist (WHO/FAO/OIE, 2017). Meanwhile, climate change is increasingly altering hydrological and temperature patterns in ways that accelerate the dispersal of waste-borne resistance determinants into new ecological zones (MacFadden et al., 2018). The contrast with what is achievable makes inaction harder to justify. Sweden’s regulatory overhaul reduced riverine antibiotic residues by 90%, demonstrating that the technical and policy solutions already exist (UNEP, 2022). On-site ozonation at treatment facilities achieves ARG removal efficiencies above 99% where it is implemented (Liguori et al., 2022). The barrier is not knowledge or technology. It is the absence of enforceable standards and the persistent treatment of pharmaceutical waste governance as an afterthought in global AMR policy (Figure 4; Tables 4, 5).

**FIGURE 4:**
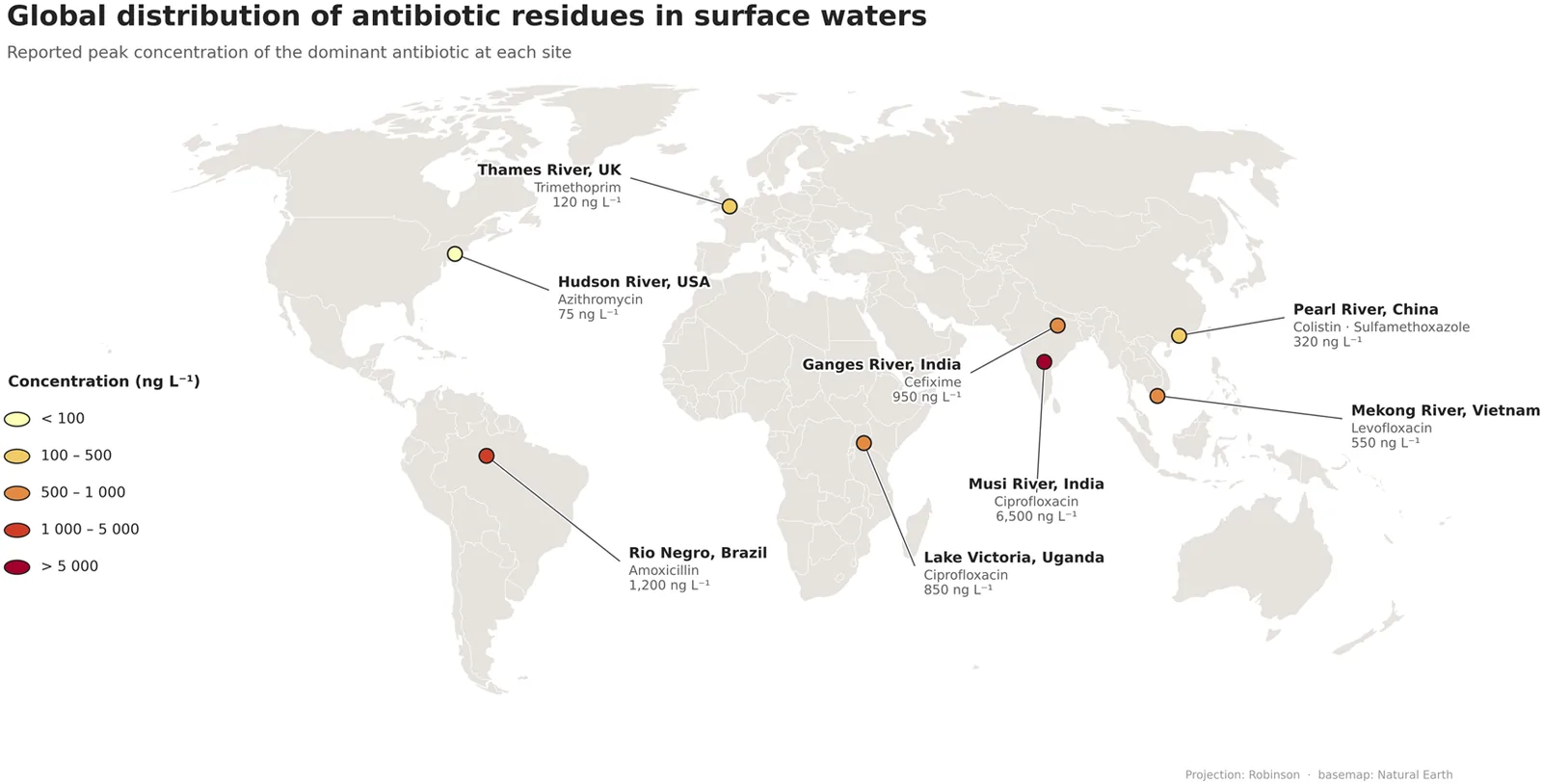
Global map of pharmaceutical waste-driven antimicrobial resistance hotspots. Shows geographical locations globally where pharmaceutical waste has significantly contributed to antimicrobial resistance (e.g., India, China, Brazil, UK, USA), based on high antibiotic residues and resistance gene prevalence (Larsson et al. 2018; Zhu et al., 2013; Larsson and Flach, 2022; Global antibiotic resistance surveillance report, 2025).

**TABLE 5:** Global pharmaceutical waste-driven AMR hotspots highlighting major antibiotic contaminants, associated resistance genes and pathogens, persistence, with one health consequences across aquatic ecosystems.

Location	Key pollutants (concentration)	Resistance genes/pathogens	Compromised antibiotic classes	Antibiotic persistence (half-life)	One Health impact	References
Musi river, India	Ciprofloxacin (6,500 ng/L)	*blaNDM-1, qnrS* (*K. pneumoniae*)	Fluoroquinolones, carbapenems	70 days (ciprofloxacin)	Poultry farms + human sewage created hybrid *blaNDM-5* superbugs	Van Boeckel et al. (2015)
Lake Victoria, Uganda	Ciprofloxacin (850 ng/L)	*blaTEM, qnrB* (*Aeromonas*)	Fluoroquinolones	70 days (ciprofloxacin)	Fishing communities show 3× higher resistance rates than urban areas	Kafaiya et al. (2026)
Mekong river, Vietnam	Levofloxacin (550 ng/L)	*qnrA, aac(6′)-Ib-cr* (*Shigella*)	Fluoroquinolones	50 days (levofloxacin)	Rice paddies flooded with wastewater increased diarrheal drug failure	Bich et al. (2019)
Pearl river, China	Colistin(320 ng/L) sulfamethoxazol	*mcr-1, blaCTX-M-55* (*E. coli*)	Colistin, β-lactams	80 days (colistin), 30 days (sulfamethoxazole)	Aquaculture farms spread *mcr-1* to seafood consumers	Liu et al. (2016), Zhu et al. (2013)
Rio Negro, Brazil	Amoxicillin (1,200 ng/L)	*blaKPC-2* (*P. aeruginosa*)	Penicillins, carbapenems	8 days (amoxicillin)	Hospital waste + urban sewage created XDR *Pseudomonas* clones	Martins et al. (2025)
Ganges river, India	Cefixime (950 ng/L)	*blaOXA-181* (*A. baumannii*)	3rd-gen cephalosporins	25 days (cefixime)	Religious mass bathing spreads CRAB through sediment	Ahammad et al. (2014)
Hudson river, United States	Azithromycin (75 ng/L)	*ermB, mefE* (*Enterococcus*)	Macrolides	60 days (azithromycin)	Urban runoff linked to macrolide-resistant UTIs in NYC	Leonard et al. (2018), Dinos (2017)
Thames river, United Kingdom	Trimethoprim (120 ng/L)	ESBL-E. coli, sul1	Sulfonamides	20 days (trimethoprim)	WWTPs released ESBL genes that later appeared in London ICU patients	Berendonk et al. (2015), Wellington et al. (2013)

### 4.3 Major sources and environmental entry pathways

Pharmaceutical waste finds its way into water systems through three main routes, creating unique environments that foster antibiotic resistance. First, industrial antibiotic production is a significant source of pollution. Research has shown that effluent from these facilities can contain alarmingly high levels of antibiotics for example, up to 31 mg/L of ciprofloxacin near production sites in Hyderabad (Larsson and Flach, 2022) and 1.2 mg/L of β-lactams in effluents from pharmaceutical parks in China (Zhu et al., 2013). These point sources act as hotspots for resistance, where low levels of antibiotics linger for kilometers downstream, creating constant selective pressure that encourages the survival of resistance genes like *bla*NDM-1 and *qnrS* (Lajqi Berisha et al., 2024). Next, healthcare facilities add to the mix with complex waste streams that include both antibiotics (for instance, 120–950 ng/L of carbapenems) and resistant pathogens that often evade standard wastewater treatment processes (Terzi et al., 2025). Lastly, municipal wastewater systems collect pharmaceuticals from human waste, with global studies revealing that unmetabolized fluoroquinolones found in 45%–65% of excreted doses persist at concentrations (200–500 ng/L) that can sustain resistance in aquatic microorganisms (Robicsek et al., 2006; Chia et al., 2020). These sources of contamination follow a predictable path in the environment: they travel through river networks as shown by *mcr-1* gene gradients that extend 30 km downstream from discharge points, accumulate in sediments (with *bla*KPC lasting over 180 days in Brazilian river sediments, and transfer to soil via biosolids, demonstrating a 100-fold increase in antibiotic resistance genes in fields irrigated with wastewater (Figure 4; Table 5). The result is that these environmental compartments act like bioreactors for the evolution of resistance, where low levels of antibiotics keep the selection pressure on, while aquatic biofilms boost the rates of horizontal gene transfer by 2–3 orders of magnitude (Zhang et al., 2019; Karkman et al., 2019).

### 4.4 Human exposure pathways and health implications

The shift from environmental resistance to a clinical threat happens through well-known, yet poorly monitored, exposure routes that complete the contamination cycle. Drinking water is the most direct pathway, with 40% of tested groundwater wells in India showing the presence of blaNDM at levels that strongly correlate (R^2^ = 0.73) with local hospital rates of carbapenem resistance (UNEP, 2022). The agricultural reuse of contaminated water and biosolids further spreads this issue through the food chain, as seen by the presence of ESBL *E. coli* in 28% of vegetables irrigated with wastewater across seven countries and tetM-containing bacteria found in 65% of soils fertilized with manure (Tiedje et al., 2022; Dinos, 2017). Occupational exposures also add to the risks for instance, Vietnamese aquaculture workers showed a 3.7-fold increase in gut colonization by *qnrS*-positive bacteria compared to controls (Bich et al., 2019). Additionally, recreational water users at contaminated beaches carry significantly higher levels of resistance genes (Leonard et al., 2018). Together, all these exposure routes create a troubling cycle of resistance: patients release antibiotic residues and resistant bacteria into sewage systems, where inadequate treatment allows for genetic recombination and the amplification of resistance before these pathogens re-enter human populations through various environmental channels (Karkman et al., 2019). The connection between pollution sources and human settlements creates predictable risk patterns, as shown by genomic studies revealing identical *bla*CTX-M-55 plasmids in riverine *E. coli* and clinical isolates from the same watershed (Venkatesan et al., 2023). While technological solutions like advanced oxidation processes can cut pharmaceutical loads by over 90% in targeted effluent, the lack of global discharge standards continues to fuel contamination, especially in regions where high pharmaceutical production meets inadequate waste management infrastructure (Liguori et al., 2022; WHO/FAO/OIE, 2017; MacFadden et al., 2018).

### 4.5 Clinical waste as a contributor to antibiotic resistance

Clinical waste is an important reservoir for antibiotic resistance genes (ARGs) and resistant microorganisms, spreading to the environment via a range of interlinked pathways. Clinical and healthcare facilities produce complex waste streams with residual antibiotics (e.g., 200–1,000 ng/L carbapenems in hospital effluents) viable antibiotic-resistant bacteria (ARBs), and mobile genetic elements that survive even after traditional wastewater treatment (Hart et al., 2023).

These pollutants take three main environmental pathways:

(1) Direct aqueous release of untreated or partially treated hospital wastewater into municipal sewer systems, where research shows 10–100× greater ARG abundances than domestic wastewater; (2) biosolid-mediated dissemination when wastewater sludge with resistant *E. coli* (up to 10^6^ CFU/g) and *bla* CTX-M genes is applied to agricultural soils; and (3) incineration byproducts, where incomplete combustion of medical waste releases particulate-bound ARGs that deposit within 5 km of treatment facilities (UNEP, 2022; Markkanen et al., 2023).

The persistence of clinically sourced resistance in the environment is compounded by low levels of sub-inhibitory antibiotics (10 μg/L) that exert persistent selective pressure and enhance horizontal gene transmission in biofilms. Of critical note, genomic surveillance has followed identical *bla* KPC carrying plasmids from Intensive care units isolates to downstream river sediments, establishing bidirectional resistance determinant movement between clinical and environmental reservoirs (Markkanen et al., 2023; Kubin et al., 2025). This process is further increased in low-resource environments where poor segregation of waste enables 30%–50% of untreated clinical waste to find its way into municipal landfills, providing long-term reservoirs where leachate carries ARGs into groundwater (Garneau-Tsodikova and Labby, 2016; Lajqi Berisha et al., 2024). The mixture of hospital effluents with industrial waste from pharmaceutical production in aquatic environments (evidenced in India’s Musi River and China’s Pearl Delta) creates hybrid superbugs that harbor both clinical (e.g., *vanA*) and environmental (*qnrS*) markers of resistance (Figure 4; Table 5). These results highlight the pressing need for next-generation clinical waste treatment technologies, such as on-site ozonation (which decreased ARGs by 99.7%) and *in situ* genomic monitoring of hospital sewers to capture emerging resistance threats prior to environmental dissemination (Liguori et al., 2022; Kafaiya et al., 2026).

### 4.6 Evidence linking pharmaceutical waste to clinical multidrug resistance

The emergence of MDR pathogens in clinical environments has been increasingly associated with environmental pollution due to pharmaceutical waste. Industrial effluents discharged into rivers, especially where there is concentrated drug production, serve as reservoirs for resistant bacteria (Karkman et al., 2019). For instance, investigations have identified extended-spectrum beta-lactamase (ESBL) producing *E. coli* in the Ganges River with increased concentrations of resistance genes downstream of pharmaceutical clusters (Lübbert et al., 2017). The constant emission of antibiotic residues creates selective pressure such that resistant populations prevail in aquatic environments. This contamination of the environment also carries over to the food supply, with direct implications for human health (Tiedje et al., 2022). Scientific studies have isolated MDR bacteria, such as carbapenem-resistant Enterobacteriaceae, from vegetables that were watered with contaminated water and seafood caught from rivers contaminated with pharmaceutical waste. This reveals that pharmaceutical waste does not get stuck in the water systems but finds its way into human exposure routes via food, with more chances of colonization and infection by resistant pathogens (Gigliucci et al., 2025). The health implications for humans are already apparent in populations surrounding pharmaceutical manufacturing facilities (Kubin et al., 2025). Epidemiological studies have linked New Delhi metallo-beta-lactamase-1 (NDM-1)-producing infections in Indian patients to environmental contamination, specifically through water. Such populations tend to experience increased rates of untreatable infections because resistant bacteria circulate in their environment (Walsh et al., 2011; Ahammad et al., 2014). The clinical consequences are serious, as infection by such environmental MDR strains often defies even last-resort antibiotics and results in worse patient outcomes. Collectively, these results show a direct link from industrial antibiotic contamination to clinical resistance. In the absence of tighter controls on pharmaceutical waste and better sanitation infrastructure, environmental MDR bacteria will continue to fuel the global antimicrobial resistance epidemic (Global Antibiotic Resistance Surveillance Report, 2025). A One Health strategy combining environmental, agricultural, and medical interventions is needed to break this cycle and safeguard public health (MacFadden et al., 2018).

### 4.7 MDR driven by pharmaceutical and clinical waste: evidence and policy recommendations

There is growing evidence that environmental contaminants originating from pharmaceutical and clinical waste are entering the broader drug life cycle, facilitating the emergence and persistence of multidrug resistance (MDR) pathogens in hospital and community settings (Kubin et al., 2025). Multiple studies indicate that antibiotic residues and resistant bacteria released from manufacturing facilities exert sustained selective pressure within aquatic systems, promoting the proliferation and exchange of resistance genes that ultimately re-enter human populations (Zhang et al., 2019; Hart et al., 2023). For instance, investigations of major pharmaceutical manufacturing regions have revealed fluoroquinolone concentrations at ecologically concerning levels in regional water bodies, alongside high prevalence of quinolone-resistant *E. coli* in nearby clinical environments (Robicsek et al., 2006; Fick et al., 2009). These environmental–clinical linkages are reinforced by genomic analyses demonstrating identical resistance determinants in environmental and hospital isolates, confirming the bidirectional pathways of transmission (Bengtsson-Palme et al., 2018). Food chains constitute another critical route for the dissemination of resistance. In Bangladesh, ESBL-producing bacteria carrying resistance genes have been isolated from water sources adjacent to pharmaceutical industrial zones and from farmed fish and vegetables in these areas (Sintondji et al., 2023; Kawsar, et al., 2026). Similarly, studies from India reported carbapenem-resistant Enterobacteriaceae on vegetables irrigated with contaminated water, suggesting a direct route for community exposure to MDR pathogens (Karkman et al., 2019; Dinos, 2017; Reichert et al., 2021). Environmental exposure of this kind has been associated with long-term gut colonization, which increases vulnerability to subsequent infections by resistant organisms. The persistence of such transmission pathways is closely tied to uneven regulatory frameworks governing pharmaceutical discharge (Lübbert et al., 2017). While the European Union has introduced environmental quality standards for pharmaceuticals, most antibiotic production occurs in Asia, where effluent discharge limits are either absent or weakly enforced. Field investigations of pharmaceutical manufacturing sites in India have documented the discharge of untreated or inadequately treated effluents containing antibiotics at concentrations sufficient to select for resistant bacteria (Changing Markets Foundation, 2018). This regulatory asymmetry may inadvertently encourage outsourcing of production to regions with less stringent environmental oversight, effectively transferring both pollution and resistance risks across borders. The pharmaceutical sector has relied heavily on voluntary initiatives to address this challenge (Access to Medicine Foundation, 2026). While major manufacturers participate in collaborative efforts such as the AMR Industry Alliance, independent assessments have documented inconsistencies between stated standards and actual wastewater management practices (UNEP, 2022). Such gaps underscore the need for more rigorous oversight and binding environmental discharge standards (AMR Industry Alliance, 2023; Halawa et al., 2024) (Table 6).

**TABLE 6:** Policy and pharmaceutical industry failures driving the environmental antimicrobial resistance crisis.

Factor	Pharmaceutical sector	Clinical consequences	References
Regulatory gaps	No global limits on antibiotic discharge; regulation on the hospital and clinical discharge is weakly enforced in Asia vs. the EU. Many major production hubs, such as those in India and China, lack modern effluent treatment plants (ETPs) or have outdated systems	MDR pathogens spread via water/food, increasing hospital-acquired infections	Bengtsson-Palme and Larsson (2016), Karkman et al. (2018), Lübbert et al. (2017), WHO/FAO/OIE (2017)
Greenwashing	Voluntary industry standards lack transparency; effluent violations are common	Resistant strains from polluted environments enter healthcare systems	Changing Markets Foundation (2018), Access to Medicine Foundation (2026)
Economic pressures	Cost-cutting in generic drug production is leading to poor waste treatment	Cheap antibiotics fuel environmental AMR, reducing treatment efficacy	Jonas et al. (2017)

## 5 The One Health continuum: ecological connectivity between environmental, animal, and human resistome

The One Health framework recognizes that human health, animal health, and environmental integrity are not parallel concerns but deeply entangled systems whose boundaries are defined by biological convenience rather than ecological reality. Nowhere is this entanglement more consequential than in the dissemination of antimicrobial resistance. The movement of resistance determinants across the environmental, agricultural, and human domains does not follow linear transmission chains but rather operates as a continuous, bidirectional ecological exchange being one that pharmaceutical and clinical waste contamination actively accelerates. Understanding this continuum at the mechanistic level is essential for explaining why resistance persists and amplifies even in the absence of direct antibiotic pressure, and why clinical containment efforts consistently fail without addressing their environmental foundations (WHO/FAO/OIE, 2017; Djordjevic et al., 2024).

### 5.1 The environmental resistome: an ancient and expanding reservoir

A foundational but frequently overlooked principle is that antibiotic resistance did not originate with the clinical use of antibiotics. Natural microbial communities in soil, sediment, and water harbor a vast diversity of resistance genes that predate pharmaceutical production by millions of years, having evolved as competitive and defensive tools in microbial ecosystems (D’Costa et al., 2011; Nesme and Simonet, 2015). This pre-existing collection of resistance determinants collectively termed the environmental resistome, constitutes the genetic reservoir from which clinically relevant resistance genes are increasingly recruited under modern selective pressure (Wright, 2010). The critical implication is that pharmaceutical contamination does not create resistance from nothing; it selects, amplifies, and mobilizes resistance elements that are already present in environmental microbial communities. Sub-inhibitory concentrations of antibiotics discharged from manufacturing facilities, hospitals, and agricultural operations function as powerful evolutionary filters, enriching those bacterial populations that carry resistance genes and facilitating their transfer onto mobile genetic elements that can cross species boundaries (Bengtsson-Palme and Larsson, 2016; Larsson and Flach, 2022). Studies using culture-independent approaches have documented that antibiotic-contaminated river sediments show resistome profiles markedly enriched compared to pristine upstream controls, with ARG diversity and abundance increasing in direct proportion to contamination load (Pal et al., 2015). This means that every unregulated pharmaceutical discharge event is, in ecological terms, an experiment in directed resistome expansion one whose outcomes propagate far beyond the discharge point through hydrological connectivity, sediment transport, and microbial dispersal.

A profoundly under-appreciated component of this environmental resistome is the significance of commensal organisms as key reservoirs and active agents of antibiotic resistance gene dispersal. The dominant perspective within the AMR literature, much of it also present in the clinical surveillance literature, defines the issue in terms of well-established pathogens, such as *K. pneumoniae, A. baumannii*, and the ESKAPE group, a paradigm that provides a fundamental misrepresentation of environmental resistome ecological dynamics. In contaminated aquatic environments, non-pathogenic organisms inhabiting sediment, biofilm, and water columns possess resistance determinants on MGEs-such as mobile conjugative plasmids, ICEs, and genomic islands-which they horizontally transfer to pathogens when exposed through contact with humans or animals (Wellington et al., 2013; Pendleton et al., 2013). Non-pathogenic bacteria lack the fitness disadvantages incurred during an active infection and under host immune pressure, making it possible that they constitute a more evolutionarily plastic resistome interface where ARGs may be accumulated and recombined more effectively under prolonged sub-inhibitory antibiotic pressures that might counteract potential fitness penalties in clinical strains. Recent research has begun to document this phenomenon in ecologically important non-pathogens. Investigated RND efflux pump activity in *Sphingobium yanoikuyae* P4, a non-pathogenic environmental bacterium from the Sphingomonadaceae family, demonstrating its capacity to harbour and express efflux-mediated resistance under conditions of antibiotic pollution. This is particularly relevant as *Sphingobium* and its allied *Sphingomonads* are abundant in polluted aquatic and soil habitats, are routinely exposed to both pharmaceutical wastewater and agrochemical input, and are metabolically flexible, possibly predisposed to survival in metabolically diverse environments with diverse chemicals (Bhatt et al., 2026). In a similar vein, non-clinical environmental *Pseudomonas* species, *Acinetobacter* spp. associated with soil, are accepted environmental reservoirs of ARGs in that mobile elements are evolutionarily related (phylone network and function) and physically associated with clinical isolates, demonstrating a genetic link between both environments (Nesme and Simonet, 2015; Gigliucci et al., 2025). Combined, these studies demonstrate that effective One Health surveillance should not simply focus on sentinel pathogens, but rather also systematically monitor the non-pathogenic environmental reservoir of bacteria as an essential part of the network where resistance genes move and concentrate before returning to clinical settings (Ferdinand et al., 2023).

### 5.2 Agricultural systems as ecological bridges

Livestock agriculture occupies a pivotal position in the One Health continuum, functioning simultaneously as a major recipient of antibiotic selective pressure and as an active conduit linking environmental and human resistomes (Van Boeckel et al., 2015; Tiseo et al., 2020). Globally, more antibiotics are consumed in animal husbandry than in human medicine, with a substantial proportion administered at sub-therapeutic doses for growth promotion rather than infection treatment precisely the concentration range most effective at driving resistance selection without eliminating the bacterial host (Klein et al., 2024). The ecological consequences extend well beyond the farm boundary. Livestock shed enormous quantities of resistant bacteria and ARGs in their waste which enters the same soil and water systems identified earlier in this review as pharmaceutical contamination hotspots. Manure-amended agricultural soils show ARG concentrations up to 28,000 times higher than non-amended controls, and rainfall events mobilize these genes into surface water networks where they join antibiotic residues from pharmaceutical and clinical sources (Tiedje et al., 2022). The result is a convergence zone where resistance elements of clinical, agricultural, and environmental origin co-mingle, creating opportunities for the assembly of novel multi-resistance plasmids that no single source alone would generate. Particularly alarming is the role of aquaculture, which bridges agricultural and aquatic systems in ways that are directly relevant to the contamination pathways described in Section 3. Antibiotics are widely used in fish and shrimp farming, often administered directly into pond water, creating aquatic environments under sustained antibiotic selection that are ecologically continuous with the rivers and estuaries where pharmaceutical waste accumulates (Reichert et al., 2021). As documented in Table 5, aquaculture farms using copper-contaminated feed show *mcr-1* (colistin resistance) gene abundances up to eight times higher than control sites, demonstrating how agricultural and metal contamination interact within the same ecological system to stabilize resistance in the food chain and surrounding environment (Zhu et al., 2013; Wang et al., 2023).

### 5.3 Aquatic organisms as intermediate hosts in resistance ecology

A dimension of resistance ecology that receives insufficient attention in the AMR literature is the role of non-human aquatic organisms as intermediate hosts that harbor, amplify, and transmit resistant bacteria between environmental compartments and human exposure points. Fish, shellfish, amphibians, and aquatic invertebrates are not passive bystanders in resistance ecology. They are biologically active participants whose gut microbiomes, immune systems, and ecological behaviors shape resistance dynamics in ways that purely chemical or genetic analyses fail to capture (Cabello et al., 2013; Watts et al., 2017). *Aeromonas* species, ubiquitous in freshwater environments and a recognized human pathogen in immunocompromised individuals, have been documented carrying class 1 integrons and plasmid-borne ARGs in rivers contaminated by pharmaceutical effluents, with resistance profiles that mirror those found in clinical isolates from the same catchment area mentioned in Table 5. *Vibrio* species in coastal and estuarine environments similarly accumulate resistance genes through HGT with environmental bacteria, creating reservoirs with direct food safety implications given their association with shellfish consumption (Cabello et al., 2013). ESBL-producing *E. coli* has been isolated from wild birds, fish, and invertebrates at contaminated river sites, confirming that these organisms serve as mobile vectors redistributing resistance genes across landscape scales, dynamic that hydrological models alone cannot account for (Merkx-Jacques et al., 2013; Sintondji et al., 2023). This host-pathogen ecology in aquatic systems is particularly consequential for human exposure because it creates what might be termed a biological amplification loop: resistant bacteria from pharmaceutical or clinical waste colonize aquatic organisms, which are then harvested for food or come into contact with humans through recreational water use, transferring resistance to human gut microbiomes through pathways that are currently unmonitored in most national AMR surveillance frameworks (Karkman et al., 2019). The data presented in Section 3.2.1, ESBL *E. coli* in 28% of wastewater-irrigated vegetables, and the 3.7-fold increase in gut colonization by *qnrS*-positive bacteria in Vietnamese aquaculture workers are the measurable outcomes of this biological amplification operating across the food system (Bich et al., 2019; Sintondji et al., 2023).

### 5.4 The gut microbiome as the critical interface of the One Health continuum

The final and arguably most clinically significant node in the One Health continuum is the human gut microbiome, the ecosystem where environmental resistance genes, agricultural resistant bacteria, and clinical pathogens ultimately converge within a single host. The gut is not merely a passive recipient of environmental contamination; it is an active evolutionary arena where incoming environmental bacteria interact with resident microbiota, exchange genetic material, and potentially transfer resistance determinants onto endogenous species that are far better positioned to cause clinical infection (Djordjevic et al., 2024). Quorum sensing, discussed in Section 3.3, in the context of environmental biofilms, operates with equal sophistication within the gut ecosystem. The dense microbial communities of the gastrointestinal tract create conditions ideal for conjugative plasmid transfer, with the physical proximity of bacteria, the nutrient-rich environment, and the continuous flow of new microbial inputs from food and water providing sustained opportunities for genetic exchange. Critically, the gut microbiome of individuals in communities adjacent to pharmaceutical manufacturing sites or who consume wastewater-irrigated produce shows measurably higher ARG carriage than control populations which is a way of finding that directly connects the environmental contamination pathways documented throughout this review to individual-level clinical risk (Ahammad et al., 2014; Zhao et al., 2020). This has a profound implication for how resistance of containment must be conceived. The conventional clinical framing treats gut colonization by resistant bacteria as a hospital-acquired problem, managed through infection control protocols and contact precautions. The One Health evidence base, however, demonstrates that gut resistome enrichment is already occurring in the community through environmental and dietary exposure routes, before patients ever enter healthcare settings (Pärnänen et al., 2019; Hart et al., 2023). Patients presenting to hospitals with community-acquired infections are therefore increasingly arriving with pre-enriched gut resistome that facilitate treatment failure which is a phenomenon that stewardship programs focused exclusively on in-hospital antibiotic use are structurally incapable of preventing. This is perhaps the starkest illustration of why the environmental dimension of AMR, and the waste governance failures that drive it, must be treated as foundational to clinical resistance containment rather than peripheral to it (Djordjevic et al., 2024) (Figure 5).

**FIGURE 5:**
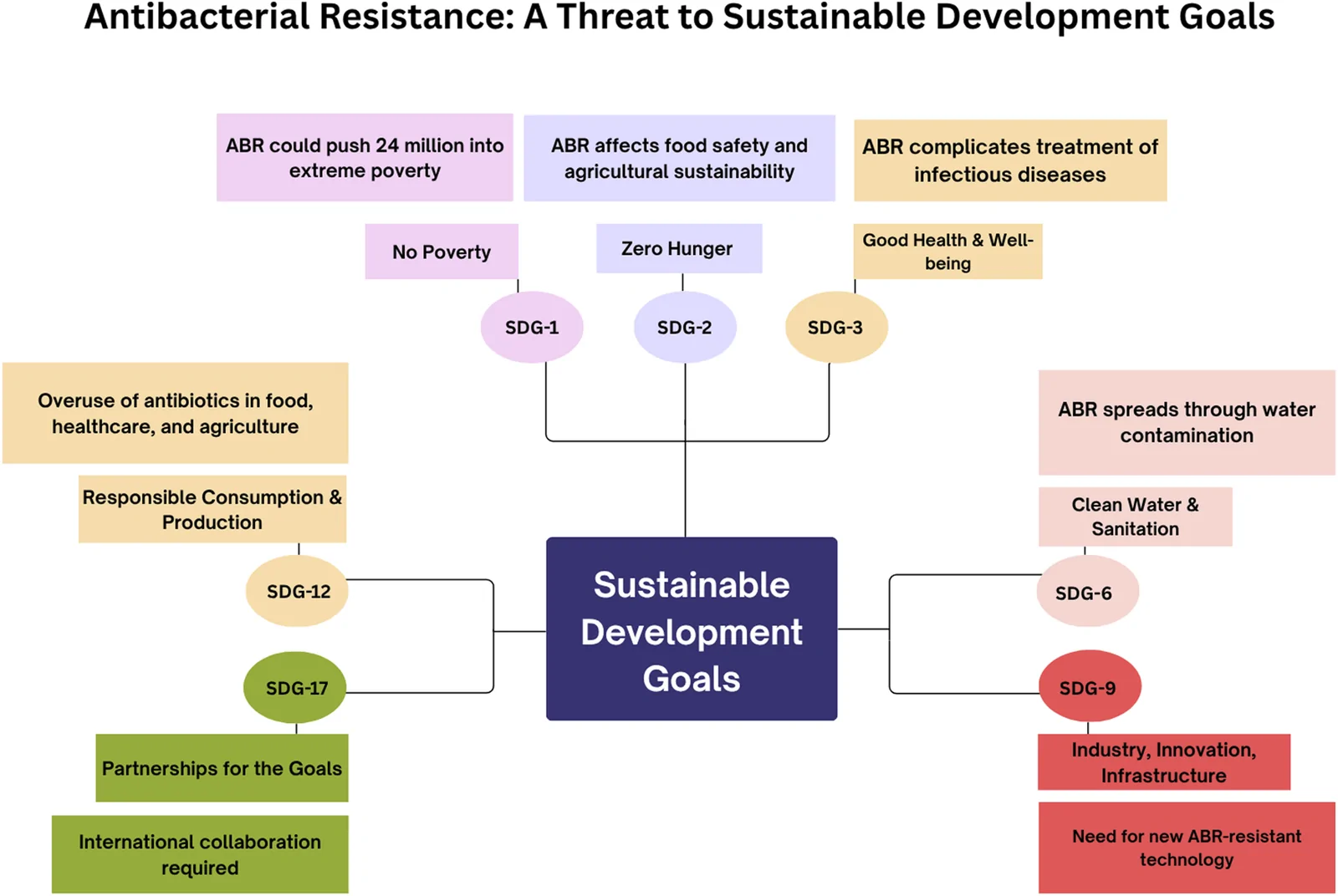
Impact of multi drug resistance (MDR) bacteria on sustainable development goals (SDGs). This figure illustrates the interlinkages between multidrug resistance (MDR) and selected united nations sustainable development goals (SDGs), namely SDG 1 (no poverty), SDG 2 (zero hunger), SDG 3 (good health and well-being), SDG 6 (clean water and sanitation), SDG 9 (industry, innovation and infrastructure), SDG 12 (responsible consumption and production), and SDG 17 (partnerships for the goals). MDR undermines multiple SDG targets by worsening health outcomes, threatening food security, increasing poverty, contaminating water systems, and constraining healthcare innovation. It also highlights the urgent need for sustainable antibiotic use and strengthened global collaboration to combat antimicrobial resistance (Laxminarayan et al., 2013; Gajdács et al., 2021; Global antibiotic resistance surveillance report, 2025; Ferdinand et al., 2023).

## 6 Sustainable development goals concerning multidrug-resistant bacteria

Antimicrobial resistance (AMR) has emerged as one of the defining systemic threats of the 21st century one that does not merely undermine public health systems in isolation but actively erodes the foundational conditions upon which sustainable development depends. The United Nations’ 2030 Agenda for Sustainable Development envisioned a world free from poverty, hunger, and preventable disease, underpinned by clean water, inclusive economies, and global solidarity. AMR threatens each of these pillars simultaneously, functioning as a cross-cutting multiplier of vulnerability across environmental, social, and economic dimensions (Tang et al., 2023). The rise of multidrug resistance (MDR) pathogens does not affect all populations equally: it concentrates its worst consequences on the most marginalized communities, amplifying existing inequalities and reversing hard-won development gains. Understanding AMR’s relationship to the SDGs is therefore not an academic exercise but a policy imperative. The following analysis examines seven Sustainable Development Goals with which AMR is most deeply entangled, drawing direct connections between the mechanisms of resistance dissemination described throughout this review and the specific development targets they place at risk (Gajdács et al., 2021; WHO, 2024).

### 6.1 SDG 1 - no poverty

The economic devastation wrought by AMR disproportionately falls on the poorest populations. Prolonged illness due to treatment-resistant infections forces families into catastrophic health expenditure cycles, depleting savings, reducing workforce participation, and pushing households below the poverty threshold. A World Bank report (Jonas et al., 2017) estimated that unchecked AMR could drive over 24 million people into extreme poverty by 2030, primarily through the compounding effects of higher treatment costs, longer hospital stays, and loss of productive life years (Kotwani et al., 2021). In low-income countries, where households already spend a disproportionate share of income on healthcare, the shift from affordable first-line antibiotics to costly last-resort therapies is often financially unsurvivable. AMR thus perpetuates and deepens the poverty traps that SDG 1 explicitly seeks to dismantle (Gajdács et al., 2021) (Figure 5).

### 6.2 SDG 2 - zero hunger

AMR undermines food security through multiple converging pathways. The heavy use of antibiotics in livestock production and aquaculture sectors that sustain the protein supply for billions selects for resistant bacteria that contaminate meat, fish, and vegetables (Van Boeckel et al., 2015). Contamination of irrigation water with antibiotic residues and resistance genes, as documented in the Ganges basin and across South Asian agricultural zones, introduces MDR bacteria directly into food crops. When farmers and agricultural workers develop resistant infections, productivity declines and food security for their communities is placed at risk. Moreover, resistant animal pathogens can decimate livestock herds, collapsing livelihoods in agro-dependent economies. The intersection of AMR with agricultural systems thus constitutes a direct threat to both the safety and the sufficiency of the global food supply (Ferdinand et al., 2023) (Figure 5).

### 6.3 SDG 3 - good health and wellbeing

It is the most directly and acutely affected goal. MDR pathogens erode antibiotic efficacy across the full spectrum of modern medicine: routine surgical procedures, cancer chemotherapy, organ transplantation, and neonatal care all depend fundamentally on effective antibiotics to prevent and treat infections. The rise of XDR and PDR strains which render carbapenems and last-resort polymyxins ineffective has already produced mortality rates exceeding 50% in some ICU settings (Coppola et al., 2022; Global Tuberculosis Report, 2024). These outcomes are concentrated in low and middle-income countries, where access to second-line treatments remains limited, surveillance infrastructure is under-resourced, and infection control capacity is weakest (Jasovský et al., 2016). The burden falls particularly on children, the elderly, and immunocompromised individuals, systematically reversing decades of progress toward universal health coverage the cornerstone of SDG 3 (Tang et al., 2023) (Figure 5).

### 6.4 SDG 6 - clean water and sanitation

It is threatened at its most basic level by the contamination of aquatic ecosystems with resistant bacteria, antibiotic residues, and mobile resistance genes. As documented extensively in Section 3, pharmaceutical manufacturing effluents, hospital wastewater, and agricultural runoff collectively transform rivers, groundwater, and drinking water infrastructure into vehicles for MDR dissemination. The persistence of resistance genes such as bla NDM-1 in groundwater wells detected at ecologically significant levels in 40% of tested sources in India illustrates how SDG 6’s targets for safe water access are directly undermined by uncontrolled AMR contamination (Reichert et al., 2021; Ahammad et al., 2014). Inadequate wastewater treatment, a defining challenge in low-income settings, allows resistance determinants to cycle continuously between human populations and water sources, sustaining exposure pathways that no amount of clinical stewardship can interrupt without parallel infrastructure investment (Ferdinand et al., 2023) (Figure 5).

### 6.5 SDG 9 - industry, innovation, and infrastructure

The AMR crisis reflects a profound failure of industrial systems to internalise the ecological costs of pharmaceutical production. Manufacturing facilities in India, China, and elsewhere routinely discharge antibiotic-laden effluents at concentrations orders of magnitude above ecological thresholds, yet only 12% of manufacturers globally test effluents for resistance genes (UNEP, 2022). Simultaneously, the pharmaceutical industry has largely retreated from antibiotic R&D due to poor commercial returns, leaving the pipeline for new antimicrobial agents dangerously thin (Ventola, 2015). SDG 9’s call for resilient infrastructure, inclusive industrialization, and innovation must therefore be applied directly to the AMR challenge: investment in advanced effluent treatment, accelerated development of novel therapeutics including bacteriophage therapy and CRISPR-based antimicrobials, and the establishment of binding environmental discharge standards are all industrial policy imperatives with direct bearing on SDG 9 targets (Gajdács et al., 2021) (Figure 5).

### 6.6 SDG 12 - responsible consumption and production

This demands urgent reform of the antibiotic life cycle from synthesis to disposal. The overuse and misuse of antibiotics across clinical, agricultural, and industrial sectors remain the primary accelerants of resistance, generating the selective pressure that transforms susceptible environmental bacteria into MDR threats. Approximately 45% of unused antibiotics are improperly discarded by households and healthcare facilities, entering waste streams without treatment (WHO/FAO/OIE, 2017; UNEP, 2022). Responsible production under SDG 12 requires manufacturers to implement lifecycle accountability: testing effluents for resistance genes, adopting closed-loop waste management systems, and adhering to stringent discharge standards. Equally, responsible consumption requires that antibiotic prescribing in human medicine, veterinary care, and food production be governed by evidence-based stewardship frameworks that match drug exposure to genuine therapeutic need, minimizing the selective pressure that resistance evolution requires (Gajdács et al., 2021; Tang et al., 2023) (Figure 5).

### 6.7 SDG 17 - partnerships for the goals

This reflects the inescapable reality that AMR cannot be solved by any single nation, sector, or discipline in isolation. Resistance genes selected by pharmaceutical discharge in one country can establish themselves clinically in another within weeks through travel and trade networks; resistant strains circulating in aquaculture in Southeast Asia contaminate seafood markets across continents. Effective AMR governance therefore demands the deepest form of international cooperation: harmonized discharge standards, equitable access to new diagnostics and therapeutics, shared genomic surveillance infrastructure, and joint R&D investment frameworks that decouple antibiotic profitability from volume of use (Jasovský et al., 2016; Global Antibiotic Resistance Surveillance Report, 2025). The Global Antimicrobial Resistance Surveillance System (GLASS) provides a foundational scaffold, but it requires substantial expansion to integrate environmental and community data streams alongside clinical ones. SDG 17’s vision of a revitalized global partnership is not merely aspirational in the context of AMR it is the minimum institutional architecture the threat demands. Taken together, these seven SDG linkages reveal AMR not as a single-sector public health problem but as a systemic sustainability crisis that cuts across every dimension of the 2030 Agenda. The mechanisms documented throughout this review pharmaceutical effluent contamination, clinical waste mismanagement, agricultural overuse, and the ecological amplification of resistance in aquatic systems are simultaneously mechanisms of poverty perpetuation, food insecurity, environmental degradation, and institutional failure (Tang et al., 2023). Addressing AMR through a sustainable development lens demands a One Health approach that integrates human, animal, and environmental health priorities within a unified governance architecture (WHO/FAO/OIE, 2017). Critically, this means treating AMR not as an addendum to the SDGs but as a cross-cutting condition of their achievability: a world that fails to contain antimicrobial resistance will find its progress toward no poverty, zero hunger, good health, clean water, responsible production, and global partnership systematically and irreversibly undermined. By embedding antimicrobial stewardship into global sustainability frameworks with the same policy urgency as climate action or pandemic preparedness, the international community can move toward realizing the SDGs not as parallel agendas competing for resources and attention, but as deeply interconnected pathways toward a more equitable, resilient, and biologically livable future (Ferdinand et al., 2023; Gajdács et al., 2021) (Figure 5).

## 7 Research gaps in environmental surveillance and governance of antimicrobial resistance

Despite growing recognition of aquatic ecosystems as critical AMR reservoirs, fundamental gaps in surveillance design, methodology, and governance continue to undermine the global capacity to monitor and respond to environmental resistance (Pärnänen et al., 2019).

### 7.1 Geographic and temporal blind spots

Current environmental AMR surveillance is geographically concentrated on a handful of well-documented hotspots, leaving large transboundary river systems which carry resistance determinants across national borders almost entirely uncharacterized (Hart et al., 2023). Equally limiting is the predominance of cross-sectional study designs that capture single seasonal snapshots, missing the resistance surges driven by monsoon runoff, agricultural discharge cycles, and episodic industrial releases. Longitudinal frameworks tracking gene abundance, decay rates, and legacy contamination over time are critically needed (Larsson et al., 2018; Gajdács et al., 2021).

### 7.2 Methodological gaps

Most surveillance programs remain anchored to culture-based methods and surface water sampling, overlooking sediments that function as long-term repositories for ARGs with documented persistence of *bla*KPC exceeding 180 days in river sediments (Dinos, 2017). Sediment core sampling should be standardized within AMR monitoring protocols. Furthermore, despite the increasing accessibility of metagenomics and quantitative PCR, these tools remain rarely integrated into national monitoring programs (Liguori et al., 2022; Djordjevic et al., 2024). Standardized genomic pipelines with spatially stratified sampling designs would substantially strengthen early-warning capacity for emerging resistance genes (Hart et al., 2023).

### 7.3 Metal co-selection and data infrastructure

The mechanisms by which heavy metals promote resistance whether through independent SOS-mediated stress responses or co-localized plasmid selection remain incompletely resolved (Vats et al., 2022; Gillieatt and Coleman, 2024). Integrating chemical half-life data for metals and antibiotics with resistome profiling would significantly improve predictive models of resistance persistence in contaminated ecosystems. At the governance level, the absence of a standardized, open-access global environmental AMR database severely limits cross-regional comparison and evidence-based policymaking. A coordinated platform under WHO or UNEP stewardship, unifying sampling methodologies and archiving metadata, is an achievable and overdue infrastructure investment (Global Antibiotic Resistance Surveillance Report, 2025; Liu et al., 2016; Balta et al., 2025).

### 7.4 Priority research directions

Three interconnected priorities should anchor the next-generation of environmental AMR research: standardized water and sediment sampling frameworks spanning diverse hydrological contexts and seasons; systematic integration of metagenomic surveillance into national and transboundary monitoring programs; and the creation of a globally accessible AMR environmental database to support comparative analysis and coordinated policy response. Addressing these gaps would shift environmental AMR science from descriptive documentation toward the predictive, actionable intelligence that effective governance requires (Nass and Zaher, 2025; Ibitoye et al., 2026).

### 7.5 Systems biology approaches to environmental resistome surveillance

Classical microbiology and culture-based methods have produced key information about AMR, but understanding the complexity of the environment resistome will necessarily require a system-level approach. The systems biology approach (metagenomics, networks, ecological modeling and computer modeling) allows us to view the resistome as an integrated system rather than a collection of resistance determinants (qualitatively differently). Shotgun metagenomic sequencing is considered the method of choice for culture-independent detection of the extent of diversity of ARGs in aquatic environments. Using metagenomic resistome profiling with databases like CARD, ResFinder, and ARG-OAP allows the simultaneous enumeration of hundreds of different resistance gene classes without biases from culturing methods in environmental samples (Larsson and Flach, 2022; Liu et al., 2024; Nass and Zaher, 2025). Used with longitudinal data on wastewater influences and effluents, river transects and aquaculture sites, metagenomic profiling can demonstrate how the resistome flows in different compartments. However, metagenomic data is not broadly used in the topics covered in this review: most included articles use a selection of specific primer sets to detect only known targets. Systems-level views of ARGs can be obtained using network-based analytical tools. In an ARGs co-occurrence network analysis, individual resistance genes are represented as nodes, and edges indicate statistically significant co-occurrence between genes within samples. These analyses can uncover some of the architectural logic behind multi-drug resistance. Here, for example, hub genes co-occur frequently with many other ARGs and these are most likely carried on mobile genetic elements; modular groups of genes will represent individual resistance platforms, such as IncF plasmids or class 1 integrons. Importantly, network architecture does not only represent what a resistome looks like. Rather the connectability of ARG hubs in the resistome network can predict horizontal gene transfer to new bacterial hosts. The use of an ecological network modeling approach of HGT is an additional system biology priority. This includes the parameterization of models with bacterial diversity, transfer rates of plasmids and ARG genes and the range of host, sub-inhibitory concentrations of antibiotics to predict the dispersal patterns of ARG genes within bacterial metacommunity in the case of various contaminating scenarios. Such models have been used to predict relative efficiency of wastewater treatment processes to decrease the concentration of ARGs downstream, with quantitative rationale of thresholds that cannot be provided only by experimental approach (Liu et al., 2016; Larsson et al., 2018). Last, machine learning and artificial intelligence methods for surveillance of AMR constitutes a research frontier, which has also entered into a phase of growing maturity. Machine learning approaches, such as supervised learning algorithms applied on metagenomic or genomic data have been found to be capable of forecasting risks of ARG propagation from environmental sensor readings (antibiotic concentration, metal concentration, bacterial community profile), whereas natural language processing is also explored to assimilate clinical AMR surveillance reports to national and global levels. Incorporating AI-based environmental surveillance into WHO GLASS data streams can lead to a system-level, real-time understanding of resistome behavior from environmental sources to clinical contexts, consistent with and complementary to ongoing One Health efforts (Raziq et al., 2026; Ibitoye et al., 2026; Global Antibiotic Resistance Surveillance Report, 2025).

## 8 Recommendations and future directions

Reversing the trajectory of multidrug resistance requires coordinated action across clinical, environmental, agricultural, and policy domains simultaneously. Piecemeal interventions targeting individual sectors have repeatedly proven insufficient; the evidence presented throughout this review demonstrates that resistance circulates through interconnected ecological systems that demand equally integrated responses. The following recommendations are organized around the core pillars of an effective, systems-level AMR response.

### 8.1 Antimicrobial stewardship and surveillance

Robust antimicrobial stewardship programs (ASPs) remain the most immediate clinical lever available. Effective ASPs go beyond restricting prescriptions; they embed evidence-based decision frameworks into clinical workflows, ensuring appropriate drug selection, dosing, and treatment duration. Critically, shorter, targeted antibiotic courses directly reduce the quantity of antibiotic residues discharged into wastewater through excretion, creating a measurable environmental co-benefit alongside the clinical one. Stewardship must be complemented by real-time surveillance. The Global Antimicrobial Resistance Surveillance System (GLASS) provides a useful international scaffold, but it remains heavily skewed toward clinical isolate data and chronically underrepresents environmental and community reservoirs Global Antibiotic Resistance Surveillance Report, 2025). National AMR action plans must expand surveillance to include wastewater, agricultural runoff, and food chain monitoring treating these not as ancillary data streams but as early-warning systems for emerging clinical resistance (Nass and Zaher, 2025).

### 8.2 Pharmaceutical and clinical waste governance

The most direct and currently under regulated driver of environmental MDR is the uncontrolled discharge of pharmaceutical and clinical waste into water systems. Binding international effluent discharge standards currently absent despite WHO/FAO/OIE guidance are urgently needed to impose enforceable limits on antibiotic concentrations in manufacturing and hospital effluents (WHO/FAO/OIE, 2017). Voluntary industry commitments, as evidence from the AMR Industry Alliance assessments consistently shows, are insufficient substitutes for regulatory enforcement (Access to Medicine Foundation, 2026). At the facility level, hospitals and pharmaceutical manufacturers must implement advanced on-site treatment technologies. Ozonation, for instance, has demonstrated ARG removal efficiencies exceeding 99% in targeted effluent streams yet remains the exception rather than the standard. Equally, the approximately 45% of unused antibiotics that are improperly discarded by households and healthcare facilities represent a tractable policy target structured take-back programs and disposal protocols can meaningfully reduce this source (Kotwani et al., 2021; Ortúzar et al., 2022).

### 8.3 Agricultural reform and one health integration

Non-therapeutic antibiotic use in livestock and aquaculture sustains one of the largest and most poorly regulated sources of environmental selective pressure. Legislative prohibition of growth-promoter antibiotics, combined with enforceable withdrawal periods before slaughter or harvest, must be adopted globally not merely in high-income countries where such reforms have already demonstrated efficacy (Van Boeckel et al., 2015). Transition support for low- and middle-income country producers, including access to vaccines, probiotics, and improved husbandry practices, is essential to make prohibition practically viable rather than merely aspirational. A genuine One Health institutional framework one that gives environmental and veterinary health agencies co-equal standing with clinical health ministries in AMR governance is the structural prerequisite for implementing cross-sectoral surveillance and coordinated intervention. Without institutional parity, environmental AMR data will continue to be generated without being acted upon (Raziq et al., 2026).

### 8.4 Innovation in treatment and environmental remediation

The near-total retreat of the pharmaceutical industry from antibiotic research and development demands structural correction through public-private partnerships, market entry rewards, and push-pull incentive mechanisms that decouple profitability from volume of use (Ventola, 2015). Simultaneously, alternative therapeutic modalities bacteriophage therapy, antimicrobial peptides, and CRISPR-Cas-based precision antimicrobials represent genuinely promising low-resistance-potential options that merit accelerated clinical development investment (Walsh et al., 2011). On the environmental side, advanced remediation technologies including bio-nanomaterials, which can disrupt bacterial membranes through physicochemical rather than biochemical mechanisms and are therefore substantially less susceptible to resistance evolution, offer sustainable complements to conventional wastewater treatment. Integration of these approaches into municipal and industrial treatment infrastructure would address contamination at source rather than attempting to manage resistance after it has entered ecological circulation (Ortúzar et al., 2022).

### 8.5 WASH infrastructure and equity

Access to clean water, sanitation, and hygiene (WASH) infrastructure is simultaneously a human rights imperative and an AMR intervention. Poor sanitation drives infection rates upward, increasing antibiotic demand; simultaneously, inadequate wastewater treatment allows resistance genes to re-enter drinking water and food chains, completing the environmental exposure cycle documented in Section 3. Targeted WASH investment in low- and middle-income countries where pharmaceutical production, agricultural antibiotic use, and clinical AMR burden converge with the weakest infrastructure represents one of the highest-impact, most equitable interventions available (Jasovský et al., 2016).

### 8.6 Global cooperation and SDG integration

Antimicrobial resistance is structurally incompatible with national-level-only governance: a resistant strain selected by pharmaceutical discharge in one country can establish itself clinically in another within weeks through travel and trade networks. Strengthening international data-sharing platforms, harmonizing discharge regulations across jurisdictions, and embedding AMR targets into Sustainable Development Goal monitoring frameworks are prerequisites for coherent global response (Global Antibiotic Resistance Surveillance Report, 2025).

The economic case for urgency is unambiguous. Projections indicate that unchecked AMR could push over 24 million people into extreme poverty by 2030 through treatment costs and productivity losses, while threatening the surgical, oncological, and neonatal care systems that depend entirely on effective antibiotics as foundational infrastructure. Mainstreaming AMR into national development planning is therefore not a public health add-on it is a prerequisite for sustainable development itself (Jonas et al., 2017; Tang et al., 2023).

## 9 Conclusion

Antimicrobial resistance (AMR) is increasingly recognized as not only a clinical challenge but also an environmental crisis, with aquatic ecosystems serving as important reservoirs and transmission pathways for multidrug resistance (MDR) bacteria and resistance genes. This systematic review, based on evidence from 167 peer-reviewed studies, demonstrates that pharmaceutical manufacturing effluents and clinical waste are among the most significant yet often overlooked contributors to the persistence and spread of antimicrobial resistance in natural aquatic environments.

The key findings reveal that environmental dissemination of resistance is driven by a combination of interconnected mechanisms, including the continuous release of antibiotic residues, biofilm formation, horizontal gene transfer, and heavy metal-mediated co-selection. Sub-inhibitory concentrations of antibiotics, together with metals such as copper, zinc, mercury, cadmium, and arsenic, create strong selective pressures that sustain clinically important resistance determinants, including *blaNDM-1*, *blaKPC*, and *mcr-1*, even in the absence of direct antibiotic exposure. Importantly, the review also highlights that non-pathogenic environmental bacteria are not merely passive inhabitants of aquatic ecosystems but serve as significant reservoirs and carriers of antibiotic resistance genes. Through extensive horizontal gene transfer within biofilms and contaminated water systems, these otherwise harmless microorganisms can act as genetic intermediaries, facilitating the movement of resistance determinants into clinically relevant pathogens. This finding reinforces the growing recognition that environmental microbial communities function as dynamic networks of genetic exchange, where resistance traits can be maintained, reshaped, and disseminated long before they emerge as public health threats. These observations support and extend previous studies that have characterized environmental resistome as active evolutionary hotspots rather than passive sinks of contamination. Unlike many earlier reviews that examined hospital wastewater, pharmaceutical pollution, molecular resistance mechanisms, or heavy metal co-selection in isolation, the present work integrates environmental, clinical, and regulatory perspectives within a comprehensive One Health framework. By doing so, it underscores the interconnected nature of human, animal, and ecosystem health and emphasizes that effective mitigation of antimicrobial resistance requires coordinated interventions that address both environmental reservoirs and clinical sources of resistance dissemination.

Despite all these contributions, some limitations of this review are that it is restricted to English-language, peer-reviewed publications, which may have excluded relevant evidence from non-English studies and grey literature. In addition, substantial heterogeneity existed among the selected studies regarding sampling approaches, geographical representation, analytical methodologies, and resistance detection techniques, limiting direct comparisons across datasets. Most available studies were observational or cross-sectional in design, providing limited information on long-term environmental-to-clinical transmission dynamics and causal relationships. Furthermore, important geographic gaps remain, particularly in low- and middle-income regions where pharmaceutical production is expanding rapidly but environmental surveillance and waste management infrastructure remain insufficient.

Future research should therefore focus on longitudinal monitoring programs that integrate metagenomics, resistome analysis, mobile genetic element tracking, systems biology approaches, and machine-learning-based predictive tools to better understand the dissemination, persistence, and ecological interactions of resistance determinants across environmental compartments. In particular, systems-level investigations combining multi-omics datasets, including genomics, transcriptomics, proteomics, and metabolomics, could provide deeper insights into the complex regulatory networks, microbial interactions, and environmental drivers that govern the emergence and dissemination of antimicrobial resistance within aquatic ecosystems. Greater efforts are also needed to quantify direct pathways linking environmental reservoirs with human and animal infections, evaluate the effectiveness of advanced wastewater treatment technologies under real-world conditions, and investigate the combined influence of pharmaceutical pollutants, heavy metals, and climate-related environmental changes on resistance evolution. The establishment of standardized international protocols for ARG monitoring and environmental risk assessment would further strengthen global surveillance frameworks, facilitate cross-regional comparisons, and support evidence-based environmental management and One Health interventions.

The findings of this review have important implications for environmental management and policy development. Environmental monitoring frameworks should incorporate antibiotic resistance genes and mobile genetic elements as indicators of ecosystem health and pollution risk. Advanced treatment technologies, including ozonation, membrane filtration, advanced oxidation processes, and genomic surveillance systems, should be more widely implemented in pharmaceutical industries and healthcare facilities to reduce the release of resistant microorganisms and antibiotic residues into aquatic environments. In parallel, stricter regulations governing pharmaceutical disposal, clinical waste management, and industrial effluent discharge are essential to minimize environmental selection pressures that favor resistance development. Integrating AMR considerations into watershed management strategies, environmental impact assessments, and broader Sustainable Development Goal initiatives would further support coordinated One Health interventions across public health, industry, agriculture, and environmental sectors.

Overall, this review underscores that environmental systems are not merely recipients of antimicrobial contamination but active participants in the evolution and dissemination of multidrug resistance. Addressing this challenge requires a shift from isolated sector-specific responses toward integrated environmental and public health strategies that recognize the inseparable links between ecosystem integrity and human wellbeing. Without coordinated action involving improved waste management, robust environmental surveillance, technological innovation, and stronger regulatory frameworks, aquatic ecosystems will continue to serve as long-term reservoirs and amplification hubs for antimicrobial resistance, posing growing risks to global health and sustainable development.
